# High research productivity in vertically undifferentiated higher education systems: Who are the top performers?

**DOI:** 10.1007/s11192-018-2644-7

**Published:** 2018-01-27

**Authors:** Marek Kwiek

**Affiliations:** Center for Public Policy Studies, UNESCO Chair in Institutional Research and Higher Education Policy, University of Poznan, ul. Szamarzewskiego 89, 60-569 Poznan, Poland

**Keywords:** Inequality in science, Publication productivity, Lotka’s square law, Stratification in science, Reward structure, Skewed distribution, Stars, Cumulative advantage, Poland

## Abstract

The growing scholarly interest in research top performers comes from the growing policy interest in research top performance itself. A question emerges: what makes someone a top performer? In this paper, the upper 10% of Polish academics in terms of research productivity are studied, and predictors of entering this class are sought. In the science system (and Poland follows global patterns), a small number of scholars produce most of the works and attract huge numbers of citations. Performance determines rewards, and small differences in talent translate into a disproportionate level of success, leading to inequalities in resources, research outcomes, and rewards. Top performers are studied here through a bivariate analysis of their working time distribution and their academic role orientation, as well as through a model approach. Odds ratio estimates with logistic regression of being highly productive Polish academics are presented. Consistently across major clusters of academic disciplines, the tiny minority of 10% of academics produces about half (44.7%) of all Polish publications (48.0% of publications in English and 57.2% of internationally co-authored publications). The mean research productivity of top performers across major clusters is on average 7.3 times higher than that of the other academics, and in terms of internationally co-authored publications, 12.07 times higher. High inequality was observed: the average research productivity distribution is highly skewed with a long tail on the right not only for all Polish academics but also for top performers. The class of top performers is as internally stratified as that of their lower-performing colleagues. Separate regression models for all academics, science, technology, engineering and mathematics academics, and social sciences and humanities academics are built based on a large national sample (2525 usable observations), and implications are discussed.

## Introduction

The world of science has always been utterly unequal (Ruiz-Castillo and Costas 2014; Stephan 2012): the intrinsic property of science has been what de Solla Price (1963) termed “essential, built-in undemocracy” (59). Individual performance in science tends not to follow a Gaussian (normal) distribution. Instead, it follows a Paretian (power law) distribution (O’Boyle and Aguinis 2012). Distributions of different social phenomena—such as income, wealth, and prices—show “strong skewness with long tail on the right, implying inequality” (Abramo et al. 2017a: 324). Academic knowledge production is not an exception because unproductive scientists work alongside ‘top researchers’ in academic units, universities, and national systems (Abramo et al. 2013; Piro et al. 2016). In more internally competitive and vertically differentiated systems (such as Anglo-Saxon systems), top researchers tend to be concentrated in elite universities and low performers in less prestigious tiers of the system. In the Polish case of an internally uncompetitive and vertically undifferentiated higher education system, with a long tradition of equality in allocating research funding and an only emergent regime of grant-based competitive research funding from the National Research Council (created in 2011), top researchers are scattered across the whole system.

The growing scholarly interest in research top performers comes from the growing policy interest in research top performance itself—and the increasing emphasis on the role of universities in global competition. Academics are at the center of the global knowledge production and global academic enterprise (Cummings and Finkelstein 2012; Leišyte and Dee 2012; Teichler et al. 2013). Not surprisingly, a question has emerged: “What makes someone a top researcher?” (Kelchtermans and Veugelers 2013: 273). In this paper, the upper 10% of Polish academics in terms of research productivity are studied in relation to the remaining 90%. The objective of present research is to study specific characteristics of this unique class of academics: who top performers are, how they work, and what they think about academic work, and to explore the predictors of entering it, based on a large sample (2525 usable observations). While bibliometric data from international (or national) datasets are perfectly suited for research productivity analyses—they can hardly be used in determining the individual characteristics of top performers, for which large-scale survey data work better.

The paper is structured as follows: Sect. 2 presents the theoretical framework, and Sect. 3 presents data and methods. Section 4, focused on the results, includes four subsections: an overview of top performers, patterns of individual research productivity and the national research output, bivariate analysis, and logistic regression analysis. The subsection on bivariate analysis consists of two parts: the first is about research productivity and working time distribution, and the second about research productivity and academic role orientation; the logistic regression analysis subsection consists of procedures and variables in the model and statistically significant individual and institutional variables. Section 5 presents the discussion and conclusion.

## Theoretical framework

Three quotations from the last half century show roughly the same phenomenon in science: “the majority of scientific work is performed by a relatively small number of scientists” (Crane 1965: 714), “no matter how it is measured, there is enormous inequality in scientists’ research productivity” (Allison 1980: 163); and most recently, “inequality has been, and will always be, an intrinsic feature of science” (Xie 2014: 809; see MacRoberts and MacRoberts 1982). The skewed distribution of scientific output found first by Lotka (1926) and shown by Price (1963) was that about 6% of publishing scientists produce half of all papers (Lotka’s law, or the inverse square law of productivity, states that the number of scientists producing *n* papers is 1/*n*^2^ of those producing one paper; see Kyvik 1989; Bensman and Smolinsky 2017). The relative importance of scientists in the right tail of the output distribution—increasingly termed *stars* recently—has endured over time (Agrawal et al. 2017: 1). The superstar effect refers to markets (“relatively small numbers of people earn enormous amounts of money and dominate the activities in which they engage” Rosen 1981: 845), and the Matthew effect (Cole and Cole 1973; Merton 1968) refers to the science system: a small number of scholars produce most of the works, attract huge numbers of citations, hold prestigious academic positions, and form the disciplines’ identity (Cortés et al. 2016; Serenko et al. 2011). For Robert K. Merton and Sherwin Rosen, performance determines rewards. In Rosen’s “economics of superstars,” small differences in talent translate into a disproportionate level of success. However, Rosen emphasizes innate talent, and Merton emphasizes external resources (DiPrete and Eirich 2006). Resources and the motivation to publish flow to scientists with high esteem in the scientific community, and that esteem “flows to those who are highly productive” (Allison and Stewart 1974: 604). Cumulative advantage is a general process by which “small initial differences compound to yield large differences” (Aguinis and O’Boyle 2014: 5). Consequently, Merton’s Matthew effect in the system of science inevitably leads to haves and have-nots, or inequalities in resources, research outcomes, and monetary or non-monetary rewards (Xie 2014; for a cross-national study of high research productivity and academic salaries in Europe, see Kwiek 2017a).

In the tradition of the sociology of science, recognition comes from scientific output (Cole and Cole 1967), and the reward system is designed to give recognition and esteem to the scientists who have best fulfilled their roles. In Merton’s (1973: 297) formulation, “the institution of science has developed an elaborate system for allocating rewards to those who variously lived up to its norms”. The reward system reinforces research activity. Few scientists will continue to engage in research if they are not rewarded for it (Cole and Cole 1967). Academics publish their work in exchange for scientific recognition. As Hagstrom (1965: 168) stated in his theory of social control in science, “recognition is given for information, and the scientist who contributes much information to his colleagues is rewarded by them with high prestige”. In this sense, research high performance (as opposed to low performance) leads to recognition in science.

The accumulative advantage hypothesis (Cole and Cole 1973) generalizes the Matthew effect to include productivity, as well as recognition: the process consists of two feedback loops in which recognition and resources are intervening variables (Allison and Stewart 1974). However, there is also the darker side of the accumulation of rewards: it is “the accumulation of failures—the process of ‘accumulative disadvantage’” (Cole and Cole 1973: 146). As scientific productivity is heavily influenced by the recognition of early work, the skewed distribution of productivity and subsequent rewards also results from the poor getting poorer. In Merton’s reputation-and-resources model of scientific careers, resources are not simply a reward for past productivity. They are a mechanism to stimulate future productivity: “the scientific community favors those who have been most successful in the past” (DiPrete and Eirich 2006: 282; Hermanowicz 2006).

Scientific productivity is skewed, and its skewness has been widely studied in terms of two standard measures of individual performance: publication numbers and citations of publications (Albarrán et al. 2011; Carrasco and Ruiz-Castillo 2014; Ruiz-Castillo and Costas 2014). In a study of 17.2 million authors and 48.2 million publications in Web of Science, Ruiz-Castillo and Costas (2014) show that 5.9% of authors accounted for about 35% of all publications. The skewness of science implies, as Seglen (1992) showed for the first time, that there will always be authors with huge numbers of publications (attracting huge numbers of citations) accompanied by a number of academics who do not publish and a large fraction of uncited publications.

Scholarly interest in the skewness of science and high individual research performance has been growing exponentially in the last few years. Highly productive academics have been studied mostly intra-nationally and in single fields of knowledge (particularly in economics and psychology), sometimes also cross-nationally (see Kwiek 2016a on top performers across 11 European systems). Recent studies on high research performers—based either on publication data or citation data—include research on star scientists (Abramo et al. 2009; Yair et al. 2017), star performers (Aguinis and O’Boyle 2014), the most productive scholars, including rising stars and stars overall (Copes et al. 2012), the best versus the rest (O’Boyle and Aguinis 2012), academic stars (Long et al. 2011), productivity stars (Aguinis et al. 2016), the most prolific female scholars and female academic stars (Weir and Orrick 2013), high-performing researchers (White et al. 2012), and superstars (Agrawal et al. 2017; Serenko et al. 2011).

Methods for determining the characteristics of top performers proliferate, and they are studied as individual scientists or scientists embedded in organizational contexts, with reciprocal relationships: how they influence and how they are influenced by their organizations or collaborative networks. The skyline for star scientists (Sidiropoulos et al. 2016) is being sought: stars are those scientists whose performance cannot be surpassed by others with respect to all scientometric indexes selected. Apart from stars, the relevant studies focus on the scientific elite or the most highly cited scientists (Parker et al. 2010, 2013), top researchers (Abramo et al. 2013; Cortés et al. 2016), the academic elite (Yin and Zhi 2017), or prolific professors (Piro et al. 2016). What makes a research star is an all-pervading question in the current productivity-obsessed and number-based academic culture. The concept of top research performers in this paper is closer to that of performance stars rather than universal stars or status stars, to use the recent typology of star employees (Kehoe et al. 2016). Star performers (“a few individuals who contribute a disproportionate amount of output”) occur in all organizations, including universities. However, a star is a relative position, and identification is possible only by viewing individuals in relation to others’ productivity (Aguinis and O’Boyle 2014: 313–315; DiPrete and Eirich 2006: 282).

Faculty research productivity and its predictors (as opposed to faculty high research productivity and its predictors) have been thoroughly explored in single-nation academic literature (see Allison and Stewart 1974; Cole and Cole 1973; Fox 1983; Ramsden 1994; Shin and Cummings 2010) and rarely in cross-national contexts (exceptions include Drennan et al. 2013; Postiglione and Jung 2013; Teodorescu 2000). Although most productivity studies do not use national samples and focus on faculty from selected academic fields, especially from natural sciences, the present study uses a national sample and refers to all academic fields (except for the regression analysis section which includes a science, technology, engineering and mathematics subsample of academics).

In traditional sociological productivity studies, highly productive academics were mostly mentioned in passing (Allison 1980; Cole and Cole 1973; Crane 1965). Exceptions include big producers in de Solla Price (1963), Croatian eminent scientists in Prpić (1996) and Golub (1998). More recently, Abramo et al. (2009) studied star scientists in the context of sex differences in research productivity in Italy and Postiglione and Jung (2013) studied top tier researchers in four Asian countries. According to Abramo and colleagues (2009: 143), the (Italian) star scientist “is typically a male full professor”. However, as their work is based on Italian bibliometric data, the authors focus on sex, academic ranks, institutional types, and academic disciplines rather than predictors of becoming a star scientist. Katarina Prpić compared the scientific productivity of eminent and average scientists in Croatia and concluded that for this elite group, “homogeneity is larger and variability is smaller than in the entire research population” (Prpić 1996: 199). Postiglione and Jung (2013: 164–165) wanted to understand better “why some faculty are more prolific in research publications than others” (Postiglione and Jung 2013: 166) and studied the 10% most and least productive academics through descriptive statistics, without referring to predictors of high research productivity. For the present study, both traditional sociological theories of social stratification in science and studies of highly productive academics (or stars) provide the conceptual underpinning.

## Data and methods

### Studying the determinants of individual-level high research productivity

Studying individual-level research performance in which the individual academic is the unit of analysis differs from studying patterns of research productivity across countries, institutional types, disciplines, academic ranks or gender (and over time). Two different methodological approaches in research literature for exploring individual-level high research productivity and its determinants (which cannot be done through bibliometric studies) can be distinguished: qualitative and quantitative. The first approach explores productivity through qualitative material: rankings of highly productive academics in particular academic disciplines are created, and then the academics in the top ranks are interviewed with a general research question, such as “how can they be so productive?” (Mayrath 2008: 42). Keys to high productivity are drawn from either targeted academic surveys of productive academics (seeking determinants of high research productivity) or from interviews with eminent, and prolific academics, or both (Flanigan et al. 2016; Kiewra and Creswell 2000; Martínez et al. 2011; Mayrath 2008; Patterson-Hazley and Kiewra 2013). Studies on research stars often rely on small-scale faculty surveys and analyses of selected top peer-reviewed journals, often combined with in-depth interviews. Qualitative studies based on varying numbers of conversations with highly productive academics seek to answer a general question: how do scholars become highly productive? The second approach, in contrast, explores predictors of high research productivity through quantitative material: academic profession surveys in which academic behavioral and attitudinal data are combined with publication data. In this paper, the survey-based, quantitative approach is used.

The paper seeks to contrast Polish top performers with the rest of academics, proceeding as follows: first, it identifies top performers in the sample; second, it examines their average research productivity (by several proxies) compared with that of the remaining 90% of academics, and third, it examines their share in the total research output—in all three steps, by major clusters of academic disciplines. In these introductory procedures only research productivity data are used. There is a trade off between a disadvantage of using self-reported data (rather than the Scopus or Web of Science data) and publication numbers as the only measure of research performance (rather than a combination of publications, citations, H-index or other measures used in bibliometrics) in introductory procedures—and an advantage of using individual-level data. Detailed individual-level data can be collected only through a survey instrument. Therefore, in the next set of procedures, behavioral and attitudinal data derived from survey questionnaires can be used as the paper seeks to compare the working time distribution (with average time investments in teaching, research, service, administration and other academic duties) and academic role orientation (interests lying primarily in teaching, research or both) of the two classes of academics.

Finally, the paper seeks to find odds ratio estimates by logistic regression for being in the top 10% in research productivity, with blocks of different individual and institutional variables. Blocks of individual variables include, for instance, “socialization to academia” (with such variables as intensive faculty guidance and research projects conducted with faculty), “internationalization and collaboration” (with such variables as research international in scope or orientation and collaborating domestically), and “overall research engagement” (with such variables as being a peer reviewer or being an editor of journals/book chapters). The two blocks of institutional variables are “institutional policies” (for instance, strong performance orientation) and “institutional support” (availability of research funds and supportive attitude of administration). These variables can be accessed through survey methodology only, the major drawback being the imprecise nature (compared with detailed bibliometric datasets) of self-reported productivity data.

### Strengths, limitations, and biases of the survey methodology

The analyses are based on self-declared data voluntarily provided by Polish academics. A crude measure of research productivity was used (the number of peer-reviewed articles and peer-reviewed article equivalents published during a 3-year reference period). Differences in reporting publication data can occur between academic disciplines and genders. Consequently, to different degrees, respondents “may present an untrue picture to the researcher, for example answering what they would like a situation to be rather than what the actual situation is” (Cohen et al. 2011: 404). Although self-reported publication data are not perfect, they do not seem to be subject to systematic errors (errors are random) or systematic bias (bias occurs when the errors tend to be in one direction more than the other; Spector 1981: 13). The exact formulations of the relevant questions are presented in Table 15 in Data Appendices. The survey instrument did not distinguish between different tiers of academic journals or separate top journals from others, and did not allow to study citation patterns. The impact factor of the journal and the number of citations the author received could not be analyzed. Individual research productivity could not be linked to individual institutions due to the data anonymization; it could be linked only to six major institutional types existing in Poland (such as legally defined universities, universities of technology, academies etc.). Consequently, it was not possible, for instance, to define the selectivity level of the employing institution, its geographic location, wealth, size, or current national and international ranking.

However, to strengthen the robustness of our productivity analyses, apart from peer-reviewed articles (PRA), three additional measures were used: peer-reviewed article equivalents (PRAE for short), internationally co-authored peer-reviewed article equivalents (IC-PRAE), and English language peer-reviewed article equivalents (ENG-PRAE). Publication counts were converted into article equivalents. The PRAE measure is calculated as the weighted sum of self-reported articles in books or journals (the value of 1 article equivalent), edited books (the value of 2 article equivalents), and authored books (the value of 5 article equivalents) published over the 3-year reference period. The same procedure was used in Piro et al. (2013: 309), Rørstad and Aksnes (2015: 319), Bentley (2015: 870) and Gorelova and Lovakov (2016: 11); most survey-based studies equate 4–6 articles to one full monograph. An individually provided share of peer-reviewed publications is applied to each observation (following Bentley 2015). The advantage of using the PRAE measure in this multi-disciplinary study is that it captures publishing through various outlets and does not focus on articles, leaving room for authored books (and edited books), which are still a major outlet in the social sciences and humanities in Poland. As Bentley (2015: 870) emphasizes, “using article equivalents and weighting of books more heavily reflects the relative contribution of the different publication types”, minimizing differences across disciplines. The internationally co-authored PRAE measure applies the individually provided share of publications co-authored with international colleagues, and the English-language PRAE measure applies the individually provided share of publications published in a foreign language (the language in question is predominantly English: 87.1% of Polish academics use English as their major foreign language in research). The question about the number of scholarly contributions was thus combined with the question about the percentage of peer-reviewed publications, English-language publications, and internationally co-authored publications. The conversion of publication counts into article equivalents is used in research productivity analyses (especially those focused on productivity correlates) based on survey data in order to make fairer comparisons of productivity across academic fields with dissimilar publication patterns (Kyvik and Aksnes 2015). So the PRAE measure was used to be able to explore more comprehensively cross-disciplinary differences in publication patterns between top performers and the rest of academics, and the IC-PRAE and ENG-PRAE measures were used to explore internationalization patterns in publishing research results between the two groups.

A substantial proportion of publishing in the humanities and social sciences in Poland consists of books and edited books, as opposed to publishing in natural sciences. The vast majority of Polish publications are still outside of major international datasets: for instance, out of 877,248 publications registered in the PBN (Polish Scientific Bibliography) national database for the period of 2013–2017, only 18.42% are indexed in the Web of Science Core Collection, and as many as 60,501 (6.89%) are monographs. Article equivalents were used specifically in multi-disciplinary studies involving major clusters of academic fields rather than merely science, technology, engineering and mathematics clusters. Examples include Ramsden (1994: 213), Guldbrandsen and Smeby (2005: 938), Kyvik and Aksnes (2015: 1441), Villanueva-Felez et al. (2013: 472), Piro et al. (2013: 309), Teichler et al. (2013: 146–147) and Arimoto (2011: 296); article equivalents were also used in *Scientometrics* and *Journal of Informetrics* (Kyvik 1989: 206; Piro et al. 2016: 945; Bentley 2015: 870; Rørstad and Aksnes 2015: 319). In Poland, the notion of article equivalents have been routinely used in parameterization (a Polish version of a research assessment exercise) and assessments of individual research output for about a decade: currently, a conversion system is used in which most Polish articles as well as all book chapters have a point value of 5 and Polish monographs have a value of 25.

### Methods and definitions

In this paper, Teodorescu’s (2000: 206) definition of research productivity is used: the “self-reported number of journal articles and chapters in academic books that the respondent had published in the 3 years prior to the survey”. The data come from the European Academic Profession: Responses to Societal Challenges (EUROAC) project, a sister project to the global Changing Academic Profession (CAP) project (see Carvalho 2017 for a recent overview of the CAP/EUROAC family of studies). The final data set dated June 17, 2011, created by René Kooij and Florian Löwenstein from the International Centre of Higher Education and Research—INCHER-Kassel, was used. The relatively low Polish response rate (11.22%) may have been caused by the increasing number of surveys to which the academic profession is routinely exposed (Mesch 2012). The response rate in Poland has been similar to response rates in several countries studying the academic profession in the last decade: studies in the Netherlands report 18% (de Weert and van der Kaap 2014: 121), in Canada 17% (Jones et al. 2014: 348), in the United Kingdom 15% (Locke and Benion 2011: 178), in Hong Kong 13% (Rostan et al. 2014: 25), in the Republic of Korea 13% (Shin et al. 2014: 183), and in Croatia, Austria, Switzerland, and Portugal about 10% or less (Teichler and Höhle 2013: 8). However, the absolute size of the Polish sample was between two and three times higher compared with other countries conducting CAP/EUROAC surveys (Shin and Cummings 2010; Cummings and Finkelstein 2012; Bentley and Kyvik 2013; Teichler et al. 2013; Marquina and Ferreiro 2015; Bentley 2015): as often argued, the bigger the sample, the more representative it is likely to be, provided the sample is randomly selected (Bryman 2012: 198).

No groups of academics were systematically excluded from the sampling frame (so “sampling bias” did not occur). At the time of the survey execution, there were 83,015 academics employed full-time in the public sector (43.8% females and 56.2% males; private sector academics were excluded, the sector being fully-teaching focused), including 17,683 full and associate professors (21.3%), 36,616 assistant professors (44.1%), 10,784 assistants (13.0%), and 15,013 senior lecturers and lecturers (18.1%, GUS 2011: 308–309). The sample of Polish academics was representative of the their population on such strata as gender and academic rank and included 45.2% of female and 54.8% of male academics, 22.6% of full and associate professors, 42.1% of assistant professors, 10.9% of assistants, and 24.4% of senior lecturers and lecturers. Sampling bias did not occur: no members of the sampling frame had no or limited chances for inclusion in the sample (Bryman 2012: 187). However, it is not possible to state to what extent the pool of respondents differs from the pool of non-respondents, and consequently, to state whether “non-response bias” occurs (Stoop 2012: 122). “Non-response bias” can occur when certain groups of respondents fail to respond or are less likely than others to participate in the survey or answer certain survey questions (Hibberts et al. 2012: 72) or when survey participation is correlated with survey variables (Groves 2006). However, non-response biases are only indirectly related to non-response rates: a key parameter is “how strongly correlated the survey variable of interest is with response propensity, the likelihood of responding” (Groves 2006: 670). It is conceivable, for instance, that highly productive academics are prone to refuse to participate in the survey because they are very busy; however, they may be inclined to participate in the survey because of a sense of civic (academic) duty, social norms producing a sense of obligation to provide help in the belief that this serves the common (academic) good, combined with a feeling that their answers count (Stoop 2012: 126–128).

Stratified random sampling was used to allow the resulting sample to be distributed in the same way as the population (Hibberts et al. 2012: 61–62; Bryman 2012: 192–193). A stratified sampling frame was created and two stratifying criteria were used: gender and academic position. The stratification of the sample mirrored the population stratification on the stratifying criteria, and mirrored simple random sample in every other way. Random sampling was used to obtain the elements from each stratum. The identification of members of the population in terms of the two stratifying criteria was possible due to the access to a national ministerial database of Polish academics. The survey was performed by the OPI, or the National Information Processing Institute: an invitation letter to participate in the web-based survey, with individually coded identifier, was sent in June 2010 to 33,000 academics, or all academics whose e-mail addresses were available at the national level at the time of the survey execution, two reminders were sent electronically between June 1, 2010 and July 20, 2010. (The National Information Processing Institute (OPI, see https://www.opi.org.pl/) is an interdisciplinary research institute which provides access to complex information concerning Polish science. OPI provides analyses for the two Polish R&D financing agencies: the National Research Council and the National Centre for Research and Development. It creates complex IT systems that gather information about science and higher education architecture in Poland: Polish Science Database, Research Equipment Database, and Polish Higher Education Information System, POL-on). However, the paper version of the survey was not mailed to non-respondents.

Due to the survey methodology used, two important methodological issues emerge: misreporting of self-reported publication data and their misspecification. The publication number misreporting is predominantly associated with surveys of sensitive topics: respondents may choose to answer dishonestly “due to a desire to present themselves in the best light to the interviewer or to avoid potential repercussions” (McNeeley 2012: 382). The questionnaire used was not viewed as sensitive by Polish academics (and the author received about 60 e-mails commenting on its content and structure but none about its sensitive nature). While overreporting socially desirable behavior in academia (for instance, increasing publication numbers) and underreporting socially undesirable behavior in academia (for instance, non-publishing) may be an issue (de Vaus 2002), and some level of misreporting is inevitable, Polish academics seem to have reported publication data and its proxies accurately: average responses matched expectations based on publicly available institutional-level and faculty-level productivity data by institutional types. For instance, average individual publishing rates corresponded to six major institutional types, with the highest rates for “universities” and “technical universities”, and the lowest for “academies” and “higher vocational institutions”. Specifically, high percentages of non-publishers and non-publishers in English (Table 5, “Rest” and Table 7, “Rest” and—for humanities and social sciences—“Top”) suggest that the misreporting was not an important issue.

The publication type misspecification occurs when, for instance, respondents count their working papers as peer-reviewed articles or conference papers as book chapters. The exact formulation of the productivity question was as follows: “How many of the following scholarly contributions have you completed in the past 3 years?” (Question D4), with the separate entries for “scholarly books you authored or co-authored” (D4/1), “scholarly books you edited or co-edited” (D4/2), “articles published in an academic book or journal” (D4/3), “research report/monograph written for a funded project” (D4/4), “paper presented at a scholarly conference” (D4/5) and “professional article written for a newspaper or magazine” (D4/6). However, the exact definitions were not provided, assuming their self-explanatory nature. The next question was formulated as follows (D5): “Which percentage of your publications in the last 3 years were—peer-reviewed” (D5/6), “published in a language different from the language of instruction at your current institution” (D5/1) and were “co-authored with colleagues located in other (foreign) countries” (D5/3). The questionnaire was explicit about different types of publications and, importantly, Polish academics are used to routinely counting different publication types for reporting purposes. The role of working papers in the Polish academic knowledge production is marginal because this type cannot be officially reported (or does not count in measuring productivity at any level, from individual to institutional: a national PBN database which collects all publications by Polish academics in all languages distinguished between six publication types (2013–2017): monographs (60,501), book chapters (295,023), and articles in four categories—List A of journals (161,629; with Impact Factor, listed in the Journal Citation Report), List B of journals (238,845; without Impact Factor), List C of journals (13,584; listed in the European Reference Index for the Humanities, ERIH) and articles from not listed journals (107,666).

Survey respondents marked one of twenty-one disciplines (as officially defined by the Central Committee for Academic Degrees and Titles in its act of October 24, 2005). Academics were grouped in eight clusters of academic disciplines, or eight academic fields in the Polish classification—humanities and arts, social sciences, physical sciences and mathematics, life sciences, engineering and technical sciences, agriculture, medical sciences and health-related sciences, and other disciplines (like fine arts)—that best represent the current structure of the Polish academic profession. The grouping was determined by the regulation of the Ministry of Science and Higher Education of August 11, 2011 on the classification of areas, fields, and disciplines: the eight clusters represent eight major academic fields. The total number of valid responses was 3704; however, in this research, academics from other disciplines (233 cases), those employed in the postdoctoral position of *docent* and teaching-focused lecturers (878 cases), and those whose work contract did not involve research (68 cases) were excluded. Cases from ‘other disciplines’ were useless for cross-disciplinary analyses due to their specificity, those from postdoctoral positions of *docent* (before 1990, a position between assistant professor and associate professor) and lecturers were useless for analyses of academic promotions, and teaching-only observations were useless for research productivity analyses. Finally, 2525 observations from seven major clusters of academic disciplines (268 top performers and 2257 lower-performing academics) were used for the analyses.

The subsample of academics involved in research from the seven major clusters of academic disciplines was divided into two subgroups: research top performers (or top performers henceforth), identified as academics ranked among the top 10% (cut-off points permitting, from 9.9 to 10.5%) of academics with the highest research performance in each major cluster of academic disciplines (separately). The second subgroup was the remaining 90% of academics involved in research. The distribution of the sample population by cluster and the threshold number of publications (the minimum number to be classified as a top performer) in terms of peer-reviewed article equivalents (PRAE) are presented in Table 1. The use of PRA and PRAE measures reflect a specificity of the Polish system which has traditionally supported the production of books across all academic fields (especially for the three turning points in academic careers: PhD dissertation, Habilitation, and full professorship). In the whole sample (2525 academics), there are 445 academics who produced 1 book in the period studied, 160 academics with 2 books, and 58 with 3 books; in the case of edited books, there are 242 academics who produced 1 edited book, 128 academics with 2 edited books, and 48 academics with 3 edited books. In 4 (out of 7) clusters of academic fields, the threshold number of peer-reviewed articles (PRA)—rather than equivalents (PRAE)—for top performers is zero: in HUM, SOC, ENGITECH and MEDHEALTH. Polish academics excessively produce non peer-reviewed articles, and produce a lot of books and edited books. There are 20 academics (out of 268, or 7.46%: 9 in HUM, 5 in SOC, 2 in ENGITECH and 4 in MEDHEALTH) who are top performers with zero peer-reviewed articles (PRA). However, in HUM, these 9 academics produced 38 books, 23 edited books and 108 non peer-reviewed articles. And in MEDHEALTH, these 4 academics produced 14 books, 5 edited books and 54 non peer-reviewed articles. They are highly productive, and the combination of PRA and PRAE measures is better suited to capture their productivity in the Polish context.

**Table 1:** The distribution of the sample population and the threshold number of publications (the minimum number to be classified as a top performer) in terms of peer-reviewed article equivalents (PRAE)

	All (*n*)	Research-involved (*n*_RI_)	% Research-involved	Top performers (*n*_TP_)	% Top performers (*n*_TP_): (*n*_RI_)	Threshold number of publications (PRAE)
HUM	613	595	97.1	62	10.1	24
SOC	291	275	94.5	29	10.0	25
PHYSMATH	194	189	97.4	20	10.3	16
LIFE	427	422	98.8	47	11.0	18
ENGITECH	571	558	97.7	60	10.5	18
AGRICULT	183	180	98.4	19	10.4	16
MEDHEALTH	313	307	98.1	31	9.9	20
Total	2593	2525	97.4	268	10.3	–

Top performers are examined through a bivariate analysis of the working time distribution and the teaching or research role orientation. Although bivariate analyses are limited as they do not control for other important factors that might affect research productivity (Teodorescu 2000: 203), the two selected variables have emerged as key in numerous productivity studies (Bentley 2015; Bentley and Kyvik 2013; Drennan et al. 2013; Jung 2014; Marquina and Ferreiro 2015; Shin and Cummings 2010; Kwiek 2016a). However, a study of multidimensional relationships requires a model approach, and therefore, odds ratio estimates with logistic regression of being a highly productive Polish academic are presented, following inferential analyses.

## Results

### Top performers: an overview

**Fig. 1:**
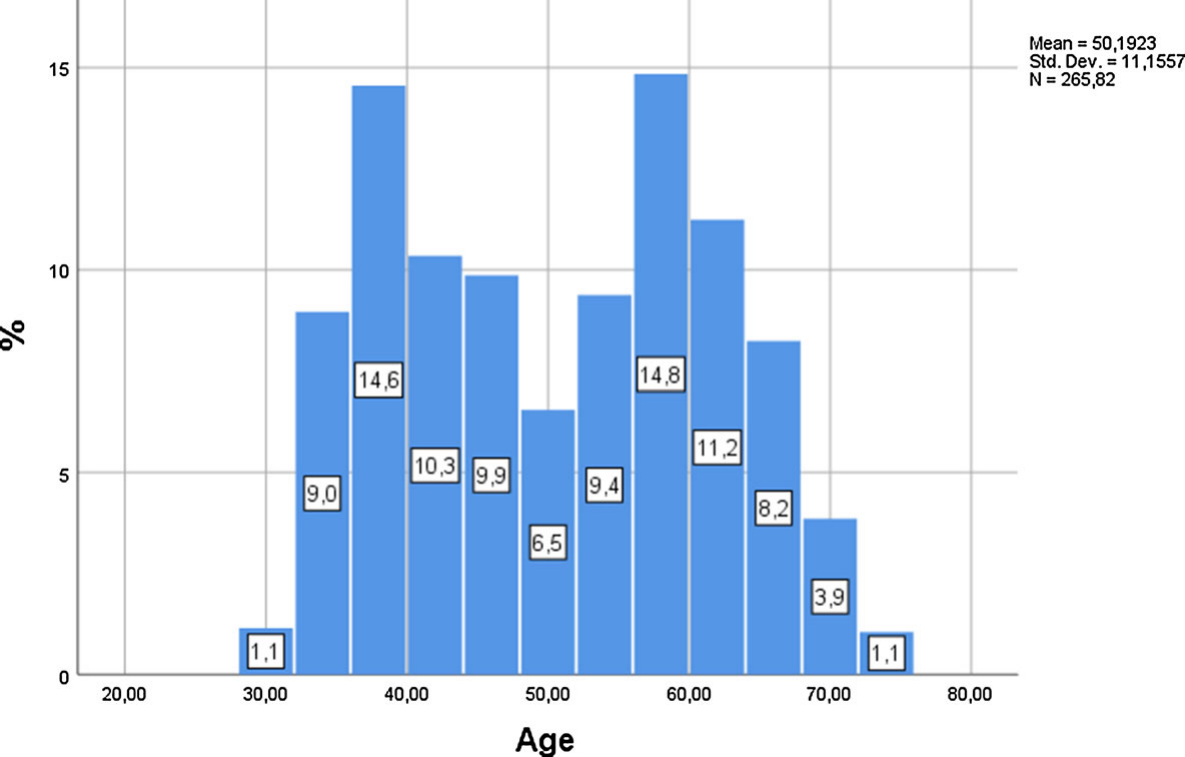
Research top performers by age group, all clusters of academic disciplines, and frequency

Frequencies of the selected demographic characteristics of the top performers are presented in Table 2. About two-thirds are men (64%), they are predominantly older (three in four is at least 40 years old, 75.3%), and almost 60% (59.8%) have at least 10 years of academic experience (calculated as working full time in the higher education sector beyond teaching and/or working as a research assistant). The mean age of top performers is 50 (standard deviation: 11.16, Fig. 1). The dominant age groups of top performers differ by academic discipline clusters. On average, the top performers are substantially younger in social sciences and the humanities and older in all other clusters (top performers aged 55 and more account for about half of the top performers in physical sciences and mathematics, engineering and technical sciences, and agriculture compared with merely one-third in the humanities and one-fourth in social sciences).

**Table 2:** Sample description: frequencies of selected demographic characteristics

	Rest (90%)		Top performers (upper 10%)		Total	
	*N*	%	*N*	%	*N*	%
Gender						
Male	1242	54.5	168	64	1410	55.5
Female	1037	45.5	95	36	1132	44.5
Age groups						
Under 30	44	1.9	2	0.6	45	1.8
30–39	854	37.4	64	24	917	36
40–49	584	25.6	62	23.3	646	25.3
50–59	414	18.1	73	27.6	488	19.1
60 and older	388	17	65	24.4	452	17.8
Academic experience*						
Under 10	688	29.8	46	17	733	28.5
10–19	662	28.7	62	23.2	724	28.1
20–29	373	16.2	58	21.8	431	16.8
30–39	423	18.3	69	25.8	492	19.1
40 and more	160	6.9	33	12.2	193	7.5
Academic disciplines						
Humanities and the arts	551	23.7	62	23.1	613	23.6
Social sciences	262	11.3	29	10.6	291	11.2
Phys sciences and math	174	7.5	20	7.4	194	7.5
Life science	380	16.4	47	17.5	427	16.5
Engineering and technical sciences	511	22	60	22.5	571	22
Agriculture	164	7.1	19	7.3	183	7.1
Medical and health sciences	282	12.1	31	11.6	313	12.1

*Academic experience means the number of years since one’s first full-time job (beyond research and teaching assistant in the higher education/research sector, Question A6)

**Fig. 2:**
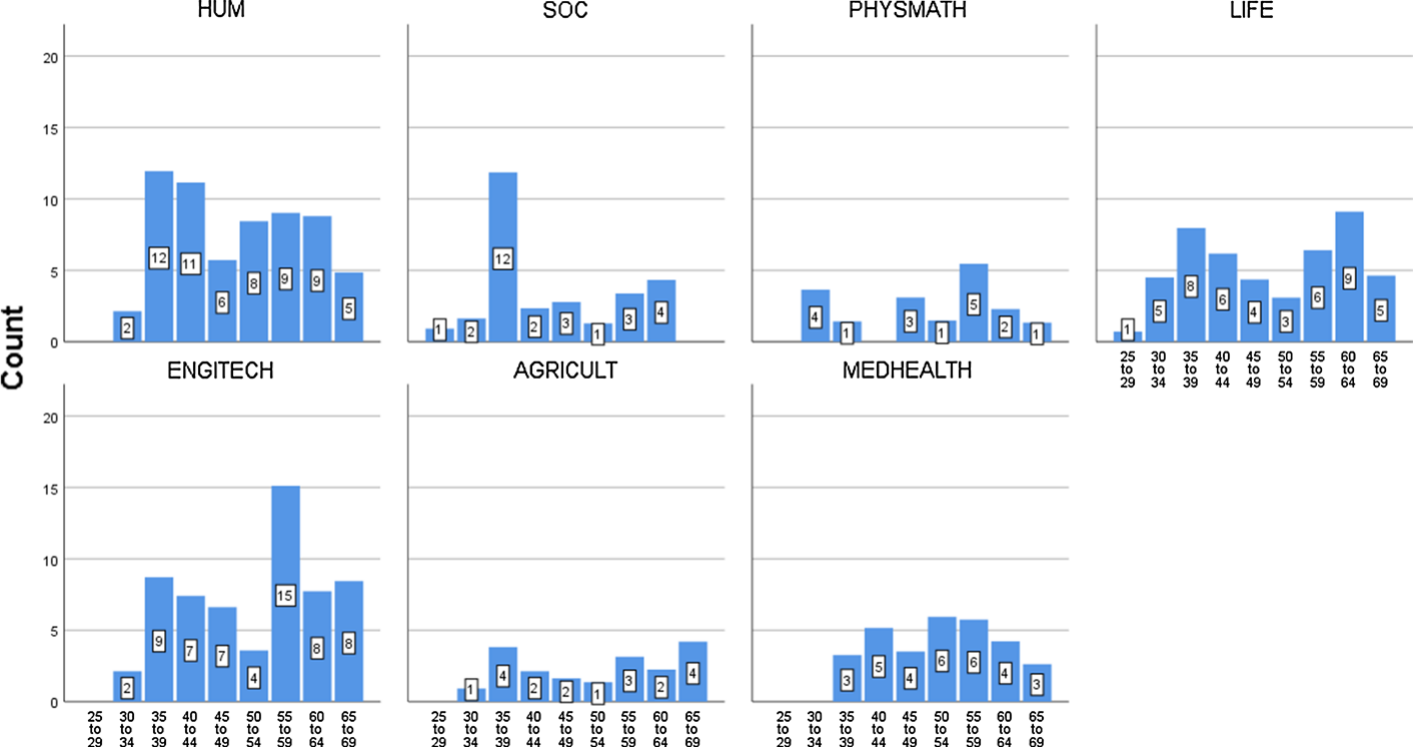
Research top performers by age group and cluster of academic disciplines, by count

A good explanation for this cross-disciplinary differentiation by age group is the deinstitutionalization of the research mission in soft fields (as opposed to hard fields) in the period of higher education expansion in 1990–2005 (Kwiek 2012, 2017b). Young top performers (an especially acute case is social sciences, with more than half of the top performers aged less than 40; see Fig. 2) were socialized in their university environment when its numerical expansion—ever-increasing enrollments—was already slowing down, leading to the current system contraction (Kwiek 2015c).

**Fig. 3:**
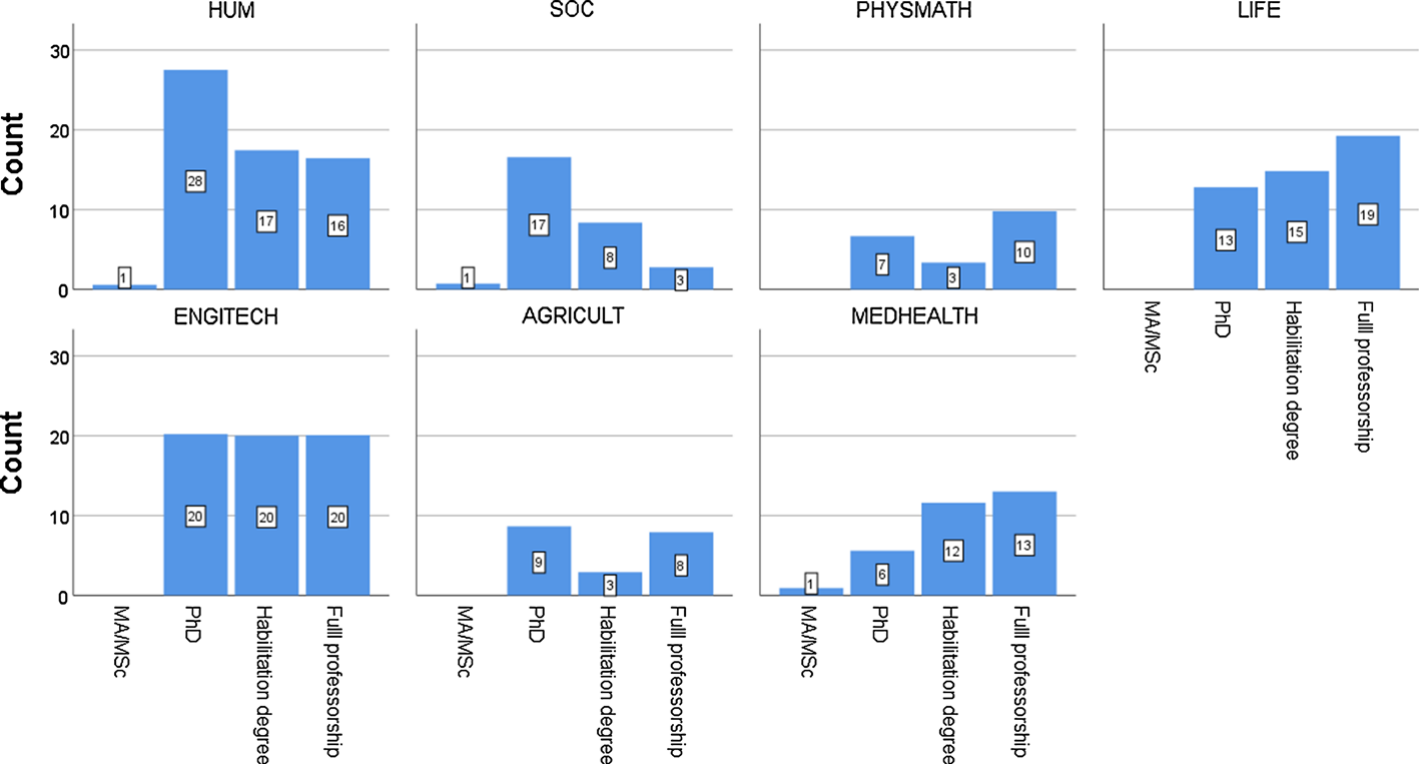
Research top performers by academic degree and cluster of academic disciplines, by count

The divide is also clear in the academic positions which top performers represent. In the soft fields, the dominant position is assistant professor (or only a PhD degree) as opposed to hard fields in which the dominant position is full professorship. Again, highly productive academics in soft fields, on average, are in lower academic positions. In hard sciences, top performers follow the pattern shown in the traditional cumulative advantage scholarly literature (Cole and Cole 1967; Merton 1968; Zuckerman 1970): the higher the position, the higher individual research productivity, or a systematic productivity increase with age (see Table 3 and Figs. 2, 3). The soft/hard divide in Polish universities is particularly strong owing to their demand-absorbing growth, turned demographically driven contraction in the last decade (Kwiek 2016b). The distribution of academics (and consequently top performers and the rest) across clusters of academic disciplines roughly corresponds to their distribution in the Polish higher education system (the tiny system of the Polish Academy of Science was excluded from data collection).

**Table 3:** Research top performers by academic degree and cluster of academic disciplines, by percentage

Degree	HUM	SOC	PHYSMATH	LIFE	ENGINTECH	AGRICULT	MEDHEALTH
MA/MSc	1.0	2.6	0.0	0.0	0.0	0.0	3.0
PhD	44.4	58.2	33.7	27.3	33.5	44.4	18.0
Habilitation degree	28.1	29.4	17.0	31.6	33.2	14.9	37.3
Full professorship	26.5	9.8	49.4	41.0	33.3	40.7	41.8

However, the statistically significant differences between top performers and other academics in terms of the speed of their academic promotion are not at the stage of studying for their doctorate or in the early academic career stage (see Table 4). The difference is that top performers receive their Habilitation degree (a second, postdoctoral degree, required in the Polish system) and then their full professorship, on average, a year faster for each degree. Answers to Question A1 in the questionnaire provided the dates of completing studies and receiving a doctoral degree, a Habilitation degree, and the professorship title, wherever applicable. Thus, the difference between the two groups is not in terms of academic promotions. The link between publishing a lot and moving up the academic ladder in Poland is weak. Full professorship is linked not only to publications but also to what is termed the “promotion of young academic cadre,” that is, the supervision of doctoral students until they graduate, which prolongs promotion to full professorship (Kwiek 2017b).

**Table 4:** The speed of academic promotion: average years between getting a degree or title

	Rest (90%)	Top performers (10%)	Significantly higher mean
Between MA/MSc and PhD	7.73	7.41	–
Between PhD and Habilitation	12.98	11.91	Rest
Between habilitation and full professorship	9.80	8.66	Rest
Between PhD and full professorship	21.12	19.66	–

Comparisons of column means (t-tests for the equality of means were performed for each academic degree, a significance level of *α* = 0.05). For each pair with a mean difference significantly different from zero, the symbol of the larger category (Top and Rest) appears in the column

Top performers compared with their lower-performing colleagues share several common features and represent a common professional profile: top performers tend to be male academics with a mean age of about 50, are full professors who collaborate more often nationally and internationally, and publish abroad more often (than the other academics). The top performers’ research tends to be international in scope or orientation, they work longer hours and longer research hours, and they are substantially more research-oriented (see Kwiek 2015a, 2017c). They focus on basic and theoretical research, (somewhat understandably) they sit on national and international committees and boards, and they are peer reviewers and editors of journals or book series more often than their colleagues (see Table 22 in the Data Appendices).

### Patterns of individual research productivity: top performers and the national research output

Detailed statistics showing average research productivity through the three article equivalent types (PRAE, IC-PRAE and ENG-PRAE) by academic disciplines cluster and by group studied (top performers vs. the other academics) is shown in Tables 5, 6 and 7 (and by peer-reviewed articles (PRA), and IC-PRA and ENG-PRA measures, are presented in Data Appendices in Tables 16, 17, and 18). By European standards, Polish academics are, on average, low research performers, and their publication outlets are largely national.

**Table 5:** Research productivity: peer-reviewed article equivalents (PRAE) published in the 3-year reference period, research top performers (10%) versus the rest (90%)

	Rest (90%)								Top performers (upper 10%)					
	Mean PRAE	95% Confidence interval, lower band	95% Confidence interval, upper band	Median	% Non-publishers	Mean PRAE for publishers only	Standard Deviation	*N*	Mean PRAE	95% Confidence interval, lower band	95% Confidence interval, upper band	Median	Standard Deviation	*N*
HUM	5.6	5.07	6.13	4.2	38.5	9.11	6.32	551	31.76	29.16	34.36	28.8	10.44	62
SOC	6.23	5.37	7.09	4	44.5	11.23	7.08	262	36.91	29.34	44.48	32	20.81	29
PHYSMATH	3.77	3.12	4.42	2	42.2	6.52	4.39	174	20.82	18.71	22.93	20	4.82	20
LIFE	3.25	2.77	3.73	0	55.8	7.35	4.81	380	27.43	24.99	29.87	25	8.55	47
ENGITECH	3.38	2.97	3.79	0	53.4	7.25	4.71	511	26.71	23.66	29.76	23.9	12.04	60
AGRICULT	3.23	2.56	3.90	0	52.1	6.74	4.38	164	25.99	21.57	30.41	24	9.82	19
MEDHEALTH	3.22	2.66	3.78	0	57.1	7.50	4.81	282	30.96	26.93	34.99	28	11.46	31

**Table 6:** Research productivity: internationally co-authored peer-reviewed article equivalents (IC-PRAE) published in the 3-year reference period, research top performers (10%) versus the rest (90%)

	Rest (90%)							Top performers (upper 10%)						
	Mean IC-PRAE	95% confidence interval, lower band	95% confidence interval, upper band	Median	% *not* intern. co-authoring	Standard Deviation	*N*	Mean IC-PRAE	95% confidence interval, lower band	95% confidence interval, upper band	Median	% *not* intern. co-authoring	Standard Deviation	*N*
HUM	0.11	0.05	0.17	0	93.4	0.67	551	1.54	0.31	2.77	0	77.1	4.93	62
SOC	0.17	0.07	0.27	0	92.5	0.82	262	1.45	− 0.21	3.11	0	87.0	4.57	29
PHYSMATH	1.24	0.86	1.62	0	68.7	2.59	174	9.59	6.22	12.96	9.6	10.7	7.7	20
LIFE	0.71	0.50	0.92	0	80.8	2.12	380	8.63	5.81	11.45	4.4	20.8	9.86	47
ENGITECH	0.35	0.24	0.46	0	86.4	1.28	511	4.35	2.57	6.13	1.45	43.5	7.04	60
AGRICULT	0.35	0.13	0.57	0	86.2	1.43	164	2	0.73	3.27	0	52.7	2.82	19
MEDHEALTH	0.23	0.10	0.36	0	90.6	1.12	282	4.88	1.76	8.00	1.15	49.4	8.87	31

**Table 7:** Research productivity: English language peer-reviewed article equivalents (ENG-PRAE) published in the 3-year reference period, research top performers (10%) versus the rest (90%)

	Rest (90%)							Top performers (upper 10%)						
	Mean ENG-PRAE	95% confidence interval, lower band	95% confidence interval, upper band	Median	% *not* publishing in English	Standard Deviation	*N*	Mean ENG-PRAE	95% confidence interval, lower band	95% confidence interval, upper band	% *not* publish. in English	Median	Standard Deviation	*N*
HUM	1.16	0.94	1.38	0	63.9	2.66	551	7.79	5.66	9.92	21.2	5.8	8.57	62
SOC	1.01	0.74	1.28	0	62.7	2.21	262	5.89	2.64	9.14	25.6	2.55	8.93	29
PHYSMATH	3.43	2.79	4.07	2	43.4	4.28	174	19.72	17.64	21.80	0.0	18	4.74	20
LIFE	2.46	2.05	2.87	0	58.6	4.09	380	21.4	18.43	24.37	0.0	20	10.4	47
ENGITECH	1.93	1.62	2.24	0	60.0	3.52	511	16.45	13.78	19.12	2.1	16.2	10.55	60
AGRICULT	1.86	1.37	2.35	0	56.9	3.2	164	11	6.92	15.08	0.0	8.08	9.07	19
MEDHEALTH	1.44	1.11	1.77	0	62.7	2.83	282	16.71	12.11	21.31	4.9	15.4	13.06	31

As can be seen in the % non-publishers column in Table 5, between 40 and 57% of Polish academics who are not top performers who conduct research are non-publishers (between 38.5% of academics in the humanities and 57.1% of academics in medicine and health-related fields did not publish a single paper or book during the reference period). As can be seen from the % not internationally co-authoring (Table 6) and % not publishing in English (Table 7) columns, their advanced internationalization in research (co-authorship as a type of collaboration) is marginal. Except for physical sciences and mathematics, about 85–95% of Polish academics who are not top performers do not co-author publications internationally; and again, except for physical sciences and mathematics, about 60% do not publish in English (Table 7).

**Fig. 4:**
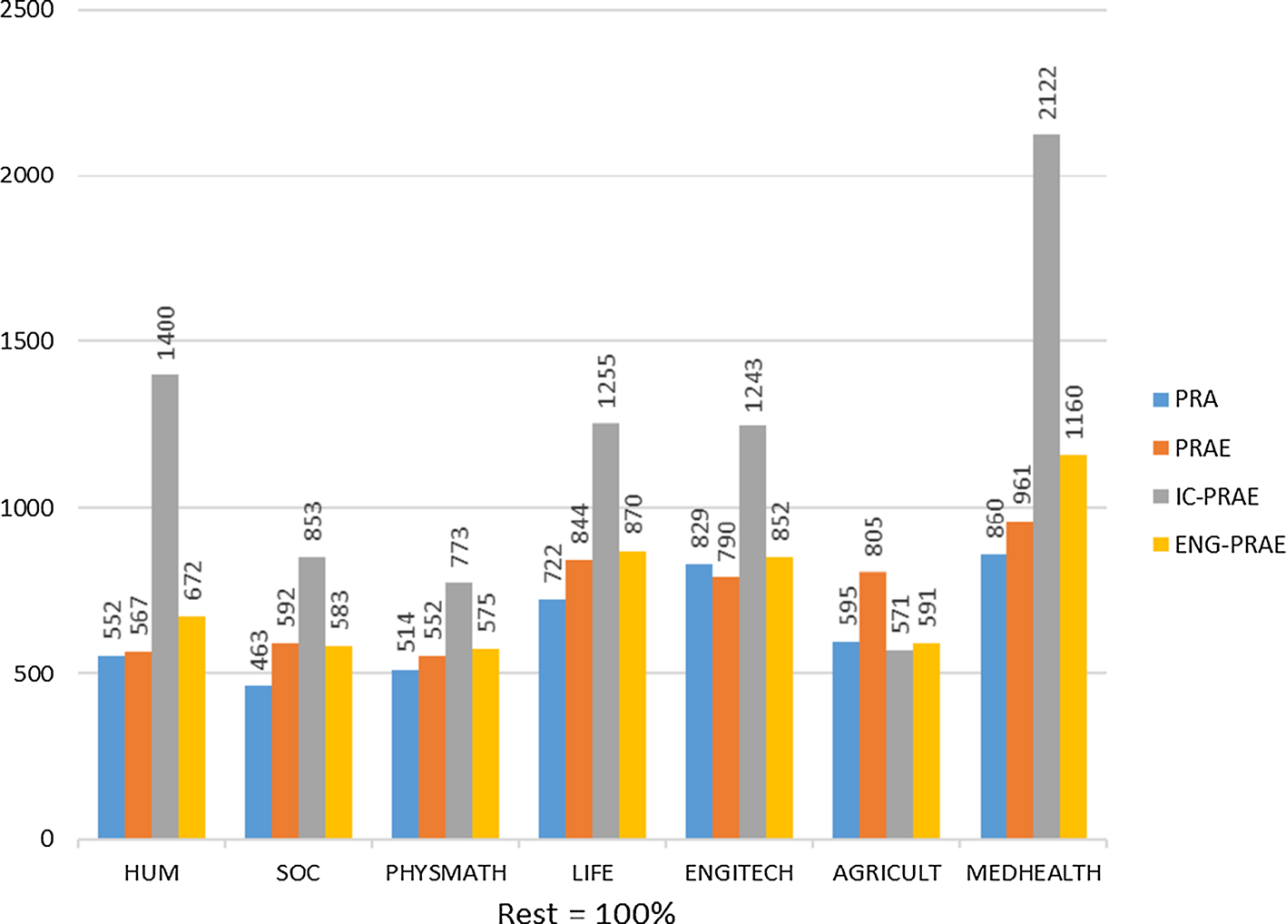
Research productivity by cluster of academic disciplines: top performers versus other academics (productivity of top performers as percentage of productivity of other academics: the Rest = 100%). The average number of peer-reviewed articles (PRA), peer-reviewed article equivalents (PRAE), internationally co-authored peer-reviewed article equivalents (IC-PRAE), and English language peer-reviewed article equivalents (ENG-PRAE) published in a 3-year reference period. For all clusters, the results are statistically significant (in %)

**Fig. 5:**
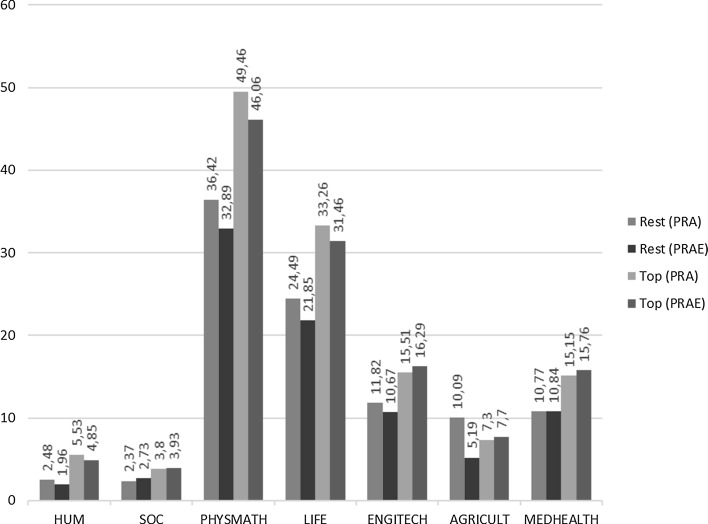
Research productivity by cluster of academic disciplines: top performers versus other academics. The percentage of IC-PRA (and IC-PRAE) in PRA (and PRAE): the percentage of the average number of internationally co-authored peer-reviewed articles and article equivalents in the average number of peer-reviewed articles and article equivalents published in a 3-year reference period. For all clusters, the results are statistically significant (in %)

The mean research productivity in terms of all measures for top performers is, on average, much higher in all clusters of disciplines: about five to eight times higher (see Fig. 4) than for the other academics. By far the biggest difference in productivity is in internationally co-authored publications (IC-PRAE)—which shows the determining role of internationalization in research for productivity: in four clusters, the difference between the two groups of academics is more than 12 times, and in three about 8 times. Interestingly, the percentage of IC-PRAE in PRAE is generally similar in all clusters (see Fig. 5): top performers produce much more, and much more with international colleagues, but there are significant cross-disciplinary variations rather than intra-disciplinary differences between the two classes of academics (with PHYSMATH and LIFE clusters with a high percentage, and HUM and SOC clusters with very low percentages, no matter which class we analyze).

Consistently across the clusters of academic disciplines, slightly less than half (44.7%) of all publications (article equivalents: peer-reviewed journal articles, book chapters, and books) come from about 10% of the most productive academics. Top performers are also responsible for about half (48.0%) of all publications in English (ENG-PRAE) and almost 60% (57.2%) of all internationally co-authored publications (IC-PRAE); the overall picture is not much different if only peer-reviewed articles are studied (see Table 8 and, in more detail, Table 19 in the Data Appendices). Strong cross-disciplinary differences are observed, however. The top performers in humanities (the upper 10.1%) produce, on average, 60.5% of all internationally co-authored publications, and in medicine and health-related fields (the upper 9.9%) about 70.5%.

**Table 8:** Average research output of Polish research top performers as a share of total research output, by cluster of academic disciplines, by productivity category, for peer-reviewed articles (PRA) and peer-reviewed article equivalents (PRAE) (in percentage)

Cluster of academic discipline/Productivity category	Share of PRAE published by top performer (%)	Share of IC-PRAE published by top performers (%)	Share of ENG-PRAE published by top performers (%)	Share of PRA published by top performers (%)	Share of IC-PRA published by top performers (%)	Share of ENG-PRA published by top performers (%)
HUM	39.3	60.5	43.4	38.6	58.6	40.9
SOC	39.8	48.6	39.3	34.1	46.1	34.3
PHYSMATH	38.8	47.2	39.8	37.0	44.4	37.2
LIFE	51.2	60.2	51.9	47.3	55.0	46.9
ENGITECH	48.4	59.6	50.4	49.6	56.4	49.3
AGRICULT	49.1	40.4	41.4	41.5	34.1	33.4
MEDHEALTH	51.9	70.5	56.6	49.1	57.9	50.2
Category mean	44.7	57.2	48.0	43.2	52.0	44.3

**Fig. 6:**
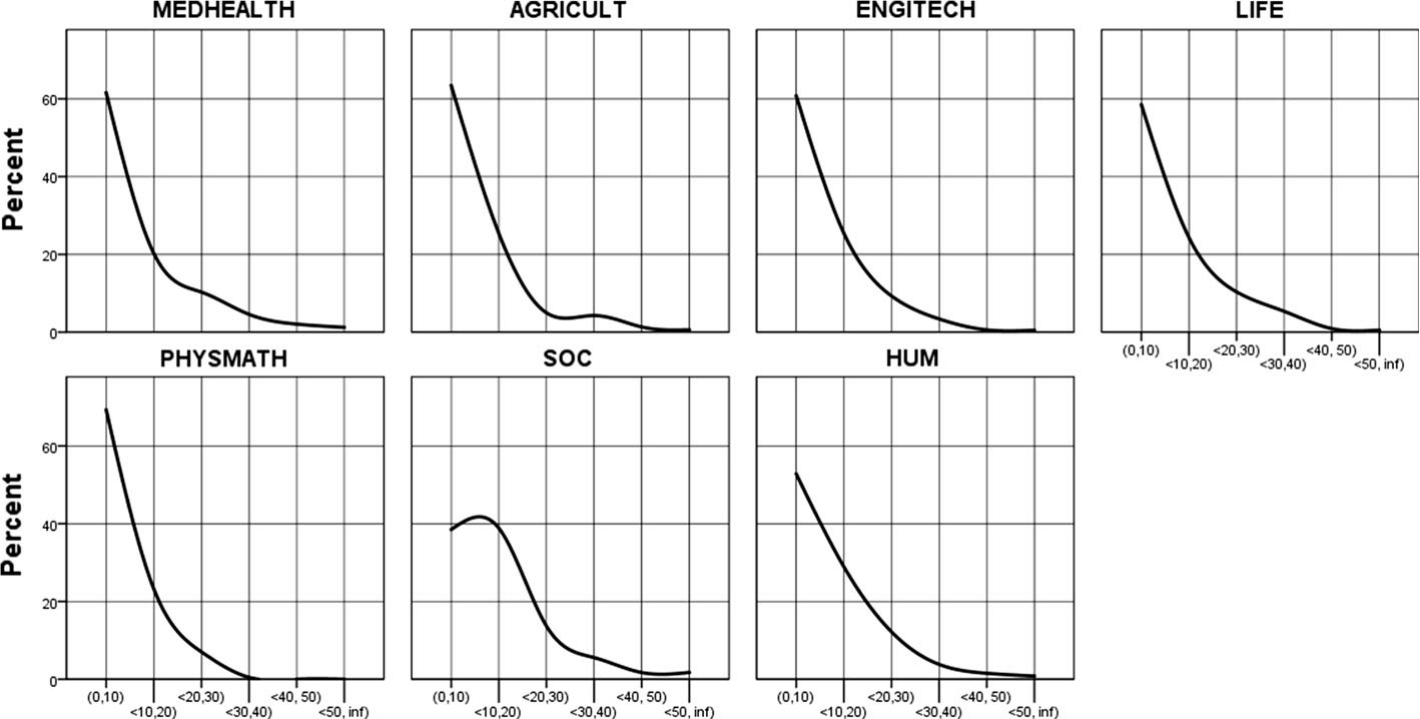
All Polish academics: the distribution of peer-reviewed article equivalents (PRAE) published during the 3-year reference period, by cluster of academic disciplines and publication number groups (in percentage). Vertically: percentage of authors, horizontally: number of papers published

**Fig. 7:**
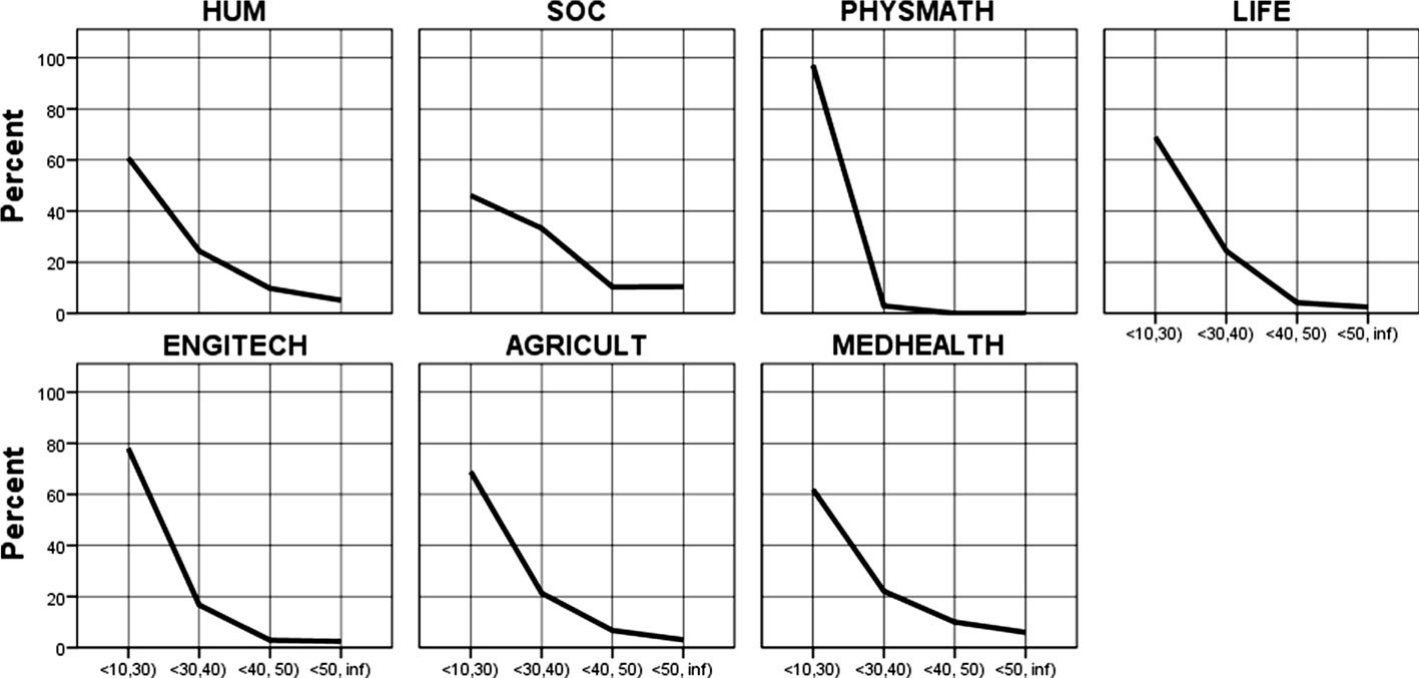
Top performers: the distribution of peer-reviewed article equivalents (PRAE) published during the 3-year reference period, by cluster of academic disciplines and publication number groups (percentage). Vertical axis: percentage of authors, horizontal axis: number of papers published

**Fig. 8:**
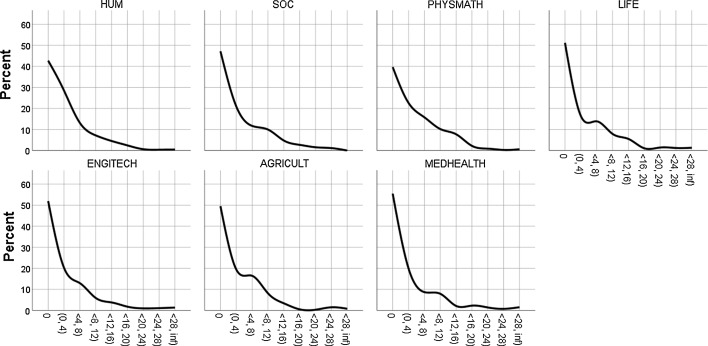
All Polish academics: the distribution of peer-reviewed article equivalents (PRA) published during the 3-year reference period, by cluster of academic disciplines and publication number groups (in percentage). Vertically: percentage of authors, horizontally: number of papers published

**Fig. 9:**
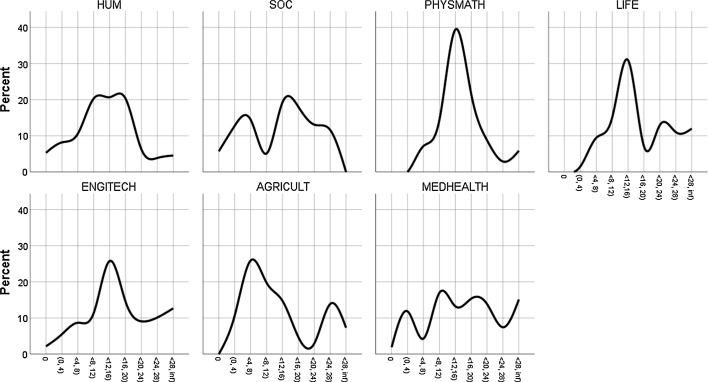
Top performers: the distribution of peer-reviewed article equivalents (PRA) published during the 3-year reference period, by cluster of academic disciplines and publication number groups (percentage). Vertical axis: percentage of authors, horizontal axis: number of papers published

The average research productivity distribution for all clusters is highly skewed to the right, not only in the case of all academics (Fig. 6) but also in the case of top performers (Fig. 7; the details in Tables 20 and 21 in the Data Appendices). Both figures show the percentage of authors on the vertical axis and the number of papers published on the horizontal axis. In the upper stratum of academics in terms of their research productivity, the productivity distribution patterns are as skewed as in the case of the lower-performing stratum; see the long tail of productivity on the right across all clusters. The upper 10% of academics is as internally stratified as the lower-performing 90%. However, this is the case only if an approach of ‘article equivalents’ is used: in the specific Polish case, in which books and edited books still significantly matter across all disciplines, the rest of academics is highly skewed but top performers are not (see Figs. 8 and 9 in Data Appendices).

### Bivariate analysis

#### Research productivity and working time distribution

Five dimensions of academic work can be captured in the dataset: teaching, research, service, administration, and other academic activities. In this paper, the focus is on the differences in the means of total working and research hours between the top performers and the other academics in each cluster of academic disciplines. The examination refers to weekly hours during the teaching periods of the academic year and the non-teaching periods. These hours are annualized, assuming that 60% for the former period and 40% for the latter period represent a good approximation for the Polish system (Bentley and Kyvik 2013 used a similar 66.6/33.3 ratio in their global study). The differences in the means for the various categories of working hours (by academic activity) between the two subpopulations are shown in Table 9. The results are based on two-sided tests that assumed equal differences in arithmetic means (with a significance level *α* = 0.05). For each pair with a mean difference statistically significantly different from zero, the symbol of the larger category (Top or Rest) appears in the column. T-tests for the equality of two arithmetic means (Top vs. Rest) were performed for each of the five types of academic activities and for each cluster of academic disciplines (Table 10).

**Table 9:** Working hour differentials by type of academic activity, academics from all discipline clusters combined. Results of t-tests for the equality of means for top performers (Top) versus the other academics (Rest)

	Mean hours per week (annualized)		T-statistics value	*P* value	Group with a significantly larger mean (Top or Rest)	% difference (Top vs. Rest)	Hours per week difference (Top vs. Rest)
	Top (upper 10%)	Rest (90%)					
Teaching	13.77	15.75	3.23	0.001	Rest	− 12.58	− 1.98
Research	22.98	18.98	− 4.49	0.000	Top	21.08	4.00
Service	5.76	5.40	− 0.84	0.405	–	6.77	0.37
Administration	7.05	6.03	− 2.36	0.018	Top	16.96	1.02
Other	5.65	5.21	− 0.77	0.442	–	8.47	0.44
Total	50.52	45.99	− 3.16	0.002	Top	− 8.97	4.53

Question B1: “Considering all your professional work, how many hours do you spend in a typical week on each of the following activities? (when ‘classes are in session’ and when ‘classes are not in session’)?” Only academics who were employed full-time and involved in teaching and research were considered (annualized mean weekly hours)

**Table 10:** Working hours differentials by type of academic activity and cluster of academic disciplines. Results of t-tests for the equality of means for top performers (Top) versus the other academics (Rest)

Cluster of academic disciplines	Academic activity	Mean hours per week (annualized)		T-statistics value	*P* value	Group with a sig. larger mean (Top or Rest)	% Difference (Top vs. Rest)	Hours difference per week (Top vs. Rest)
		Top (upper 10%)	Rest (90%)					
HUM	Teaching	14.55	15.96	0.98	0.330		− 9.69	− 1.41
	Research	21.77	20.50	− 0.63	0.528		5.83	1.27
	Service	6.04	4.84	− 1.17	0.242		19.87	1.20
	Administration	6.85	5.51	− 1.32	0.189		19.56	1.34
	Other	4.76	4.86	0.11	0.914		− 2.10	− 0.10
	Total hours	49.21	46.00	− 0.94	0.346		6.52	3.21
SOC	Teaching	19.78	17.00	− 1.39	0.167		14.05	2.78
	Research	19.09	16.09	− 1.12	0.265		15.72	3.00
	Service	6.92	6.41	− 0.25	0.805		7.37	0.51
	Administration	6.11	6.33	0.15	0.879		− 3.60	− 0.22
	Other	4.40	5.03	0.42	0.676		− 14.32	− 0.63
	Total hours	52.41	44.92	− 1.42	0.157		14.29	7.49
PHYSMATH	Teaching	11.08	13.26	1.20	0.233		− 19.68	− 2.18
	Research	35.16	22.06	− 3.85	<0.001	Top	37.26	13.10
	Service	4.65	4.13	− 0.37	0.714		11.18	0.52
	Administration	4.84	6.40	0.96	0.340		− 32.23	− 1.56
	Other	3.96	4.49	0.49	0.625		− 13.38	− 0.53
	Total hours	56.39	44.91	− 2.72	0.008	Top	20.36	11.48
LIFE	Teaching	12.29	15.57	2.34	0.020	Rest	− 26.69	− 3.28
	Research	25.67	21.64	− 2.13	0.034	Top	15.70	4.03
	Service	4.52	4.13	− 0.54	0.593		8.63	0.39
	Administration	10.12	6.47	− 3.58	<0.001	Top	36.07	3.65
	Other	6.52	4.93	− 1.26	0.210		24.39	1.59
	Total hours	53.46	47.02	− 2.25	0.025	Top	12.05	6.44
ENGINTECH	Teaching	14.07	14.74	0.58	0.566		− 4.76	− 0.67
	Research	22.09	17.33	− 2.82	0.005	Top	21.55	4.76
	Service	5.63	5.47	− 0.17	0.864		2.84	0.16
	Administration	6.31	5.79	− 0.70	0.482		8.24	0.52
	Other	6.18	5.71	− 0.46	0.643		7.61	0.47
	Total hours	50.92	45.12	− 2.20	0.029	Top	11.39	5.80
AGRICULT	Teaching	11.51	18.33	1.92	0.058		− 59.25	− 6.82
	Research	19.53	18.82	− 0.22	0.826		3.64	0.71
	Service	4.43	4.81	0.23	0.821		− 8.58	− 0.38
	Administration	5.22	6.68	0.74	0.462		− 27.97	− 1.46
	Other	7.29	5.92	− 0.66	0.514		18.79	1.37
	Total hours	44.91	51.14	1.00	0.321		− 13.87	− 6.23
MEDHEALTH	Teaching	11.73	16.13	2.31	0.022	Rest	− 37.51	− 4.40
	Research	19.26	16.03	− 1.26	0.209		16.77	3.23
	Service	8.13	7.99	− 0.07	0.947		1.72	0.14
	Administration	7.19	6.00	− 1.04	0.302		16.55	1.19
	Other	5.96	5.46	− 0.37	0.713		8.39	0.50
	Total hours	44.58	44.65	0.02	0.988		− 0.16	− 0.07

Question B1: “Considering all your professional work, how many hours do you spend in a typical week on each of the following activities? (when ‘classes are in session’ and when ‘classes are not in session’)?” Only academics who were employed full-time and involved in teaching and research were considered (annualized mean weekly hours)

The mean for the annualized total weekly working time differential between the Polish top performers and the other academics is 5 h. The Polish academia that emerges in this research is traditional: top performers, on average, spend less time on teaching-related activities (2 h per week) and more time on research (4 h per week), as well as 1 more hour on administrative duties. However, there are substantial cross-disciplinary differentials in total weekly working time, ranging from 6 h for engineering and technical sciences to as many as 12 h for physical sciences and mathematics (Table 10). In other words, Polish top performers in physical sciences and mathematics, when compared with the rest of Polish academics in physical sciences and mathematics, on average, spend an additional 69 full working days in academia per year (12 h times 46 weeks divided by 8 h per day); and more specifically, on average, they spend 13 more hours per week on research (i.e., an additional 75 days). This is the average entry ticket to the highly productive class of academics in terms of average working time allocation. A standard pattern for Polish top performers is (many) more working hours and especially, (many) more research hours (see summary of working hours differentials in Table 11).

**Table 11:** Summary: working hours differentials by type of academic activity and cluster of academic discipline

	HUM	SOC	PHYSMATH	LIFE	ENGITECH	AGRICULT	MEDHEALTH
Teaching				Rest			Rest
Research			Top	Top	Top		
Service							
Administration				Top			
Other							
Total			Top	Top	Top		

Results of t-tests for the equality of means for top performers (TP) versus the other academics (R). Question B1: “Considering all your professional work, how many hours do you spend in a typical week on each of the following activities? (when ‘classes are in session’ and when ‘classes are not in session’)?” Only academics who were employed full-time and involved in both teaching and research were considered (annualized mean weekly hours). Group with a significantly larger mean: Top versus Rest

#### Research productivity and academic role orientation

Research literature suggests that high academic productivity is correlated with high research orientation (Ramsden 1994; Shin and Cummings 2010; Teodorescu 2000). The Polish system as a whole (for all clusters of academic disciplines combined) emerges from this research as perfectly traditional. The results of the z test for the equality of fractions performed for the two subpopulations (top performers and other academics) are based on two-sided tests with a significance level of *α* = 0.05. The tests were adjusted for all pairwise comparisons within a row for each innermost sub-table using the Bonferroni correction. Z tests for the equality of fractions (Top vs. Rest) were performed for each of the four teaching and research orientation categories. Correspondingly, as before, for each pair with a fraction difference significantly different from zero, the symbol for the larger category appears in the last column (Table 12).

**Table 12:** Results of the z test for the equality of fractions, all clusters of academic disciplines combined, preferences for teaching/research (Question B2: “Regarding your own preferences, do your interests lie primarily in teaching or in research?”), research top performers versus the other academics (percent)

	Percent		Z-statistics value	*P* value	Group with a significantly larger fraction
	Top performers (upper 10%)	Rest (90%)			
Primarily in teaching	0.6	3.9	− 2.78	0.005	Rest
In both, but leaning toward teaching	15.2	28.7	− 4.63	0.000	Rest
In both, but leaning toward research	66.4	54.9	3.64	0.000	Top
Primarily in research	17.9	12.5	2.52	0.012	Top

The higher research role orientation among top performers is statistically significant, as is the higher teaching role orientation among the other academics. Top performers value research more than their lower-performing colleagues. Being interested primarily in teaching virtually excludes Polish academics from the class of research top performers: the percentage of top performers who are primarily interested in teaching is 0.6%; however, inconsistent with scholarly literature focused on the teaching-research competition (Fox 1992; Ramsden 1994; Stephan 2012; Stephan and Levin 1992), 15.2% of academics interested “in both, but leaning towards teaching” are top performers. A research role orientation is a powerful indicator of belonging to the class of Polish highly productive academics: being research-oriented is almost a statistical must, and being teaching-oriented almost excludes them from this class. However, a closer examination by clusters of disciplines is inconclusive (statistically significant results are obtained for four out of seven clusters; not reported here due to space limitations).

### Logistic regression analysis

#### Procedures and variables in the model

Differences in individual research productivity can be explained by at least three theories. The sacred spark theory (Cole and Cole 1973) states “that there are substantial, predetermined differences among scientists in their ability and motivation to do creative scientific research” (Allison and Stewart 1974: 596). Highly productive scholars are motivated by “an inner drive to do science and by a sheer love of the work” (Cole and Cole 1973: 62). Productive scientists are a strongly motivated group of researchers, and they have the stamina, “or the capacity to work hard and persists in the pursuit of long-range goals” (Fox 1983: 287; Zuckerman 1970: 241). The accumulative advantage theory developed by Robert K. Merton (1968) claims that productive scientists are likely to be even more productive in the future, while the productivity of low performers will be even lower. The accumulative advantage theory is related to the reinforcement theory formulated by Cole and Cole (1973: 114) which in its simplest formulation states that “scientists who are rewarded are productive, and scientists who are not rewarded become less productive”. Finally, according to the utility maximizing theory, all researchers choose to reduce their research efforts over time because they think other tasks may be more advantageous. As Kyvik (1990: 40) states, “eminent researchers may have few incentives to write a new article or book, as that will not really improve the high professional reputation that they already have” which may mean that “with each additional year the rewards for doing research decline” (Stephan and Levin 1992: 35). Scientists’ engagement in research can be either investment-motivated (seeking future financial rewards), consumption-motivated (solving research puzzles), or both (Thursby et al. 2007). Although the investment motive implies a decline in research productivity over one’s career, the consumption motive does not imply such a decline (Levin and Stephan 1991). A taste for science (Roach and Sauermann 2010)—that is, for non-monetary returns—causes scientists to choose academia over industry. Academics with different abilities and tastes in terms of non-monetary returns choose different careers: basic or applied research in academia or industry (Agarwal and Ohyama 2012). Time spent on research reduces current earnings but increases future earnings, as in investment models of human capital (see Kwiek 2017a on European ‘academic top earners’ in 10 countries). These three major theories of research productivity are complementary rather than competitive. To varying degrees, they are all applicable to the Polish academic profession.

An analytical model for studying high research productivity was prepared based on research literature, especially Fox (1992: 295–297), Ramsden (1994: 211–212), and Teodorescu (2000: 207). Following Ramsden (1994), it has been assumed that “any sensible explanation of research output must take into account personal (individual) and structural (environmental) factors, and preferably also the interaction between them”. Independent variables are grouped as individual and institutional characteristics in eight clusters (Table 13; the exact formulations of questions are presented in Table 15 in Data Appendices).

**Table 13:** Faculty research productivity: variables in the model (survey question numbers in parentheses)

Individual variables	Institutional variables
*Personal/demographics*	*Institutional policies*
Female (F1)	Strong performance orientation (E4)
Mean age (F2)	Research considered in personnel decisions (E6)
Full-time (A7)	*Institutional support*
PhD or lower degree (A1)	Availability of research funds (B3)
Habilitation degree (A1)	Supportive attitude of administration (E4)
Full professorship (A1)	
Work at another research institute or HEI (A8)	
Self-employed (A8)	
My academic discipline/field is important (B4)	
My institution is important (B4)	
Satisfaction with current job (B6)	
*Socialization to academia*	
Intensive faculty guidance (A3)	
Research projects with faculty (A3)	
*Internationalization and collaboration*	
Collaborating internationally (D1)	
Collaborating domestically (D1)	
Publishing in a foreign country (D5)	
Published abroad (D5)	
Research int’l in scope or orientation (D2)	
*Academic behaviors*	
Annualized mean research hours	
(60% in session and 40% not in session) (B1)	
*Academic attitudes and role orientation*	
Research-oriented (only answer 4) (B2)	
Scholarship is original research (B5)	
Basic/theoretical research (D2)	
*Overall research engagement*	
National/int’l. committees/boards/bodies (A13)	
A peer reviewer (A13)	
Editor of journals/book series (A13)	

All category variables were dichotomized through a recoding procedure. Forty-nine personal and institutional characteristics grouped in eight clusters were selected. Then Pearson Rho correlation tests were conducted to find significantly correlated predictors of the dependent variable. The predictors were entered in a four-stage logistic regression model. Multicollinearity was tested using an inverse correlation matrix, and no independent variables strongly correlated with others were found. On the main diagonal of an inverse correlation matrix, there are values without unequivocal interpretation; however, they show how strongly a given variable is correlated with all other variables. The interpretation is performed in such a way that all variables with diagonal values higher than 4 are removed from analysis (see an inverse correlation matrix in Table 23 in the Data Appendices). In addition, principal component analysis (PCA) was performed to determine whether any variables, due to their high level of correlation, could be grouped into homogenous groups. No significant interdependence between any of the variables was found. Separate models for all academics combined, science, technology, engineering, and mathematics (STEM) academics, and social sciences and humanities (SSH) academics were built. The predictive power of the fourth model (as measured by Nagelkerke’s R^2^) was the highest for STEM academics and was 0.381 (shown in Table 14). In this table, the results for the final, fourth model are presented.

**Table 14:** Odds ratio estimates by logistic regression for being in the top 10% in research productivity (STEM academics only: the core STEM/10% model)

Nagelkerke’s R2	0.381
*Individual predictors*	
Personal/demographics	
Female	
Age	
Full-time	
PhD or lower	
Habilitation degree	
Full professorship	
Work at another research institute or HEI	
Self-employed	
My academic discipline/field is important	
My institution is important	
Satisfaction with current job	
Socialization to academia	
Intensive faculty guidance	
Research projects with faculty	
Internationalization and collaboration in research	
Collaborating internationally	7.02**
Collaborating domestically	
Published abroad	7.855***
Research international in scope or orientation	0.508*
Academic behaviors/and working time allocation	
Mean research hours	1.038**
Academic attitudes and role orientation	
Research-oriented	2.333*
Scholarship is original research	
Basic/theoretical research	
Overall research engagement	
National/intern. committees/boards/bodies	
A peer reviewer	
Editor of journals/book series	3.138*
*Institutional predictors*	
Institutional policies	
Strong performance orientation	
Performance-based resource allocation	
Institutional support	
Availability of research funds	
Supportive attitude of administration	
Constant	0.006***

Results not statistically significant are not shown in the table

***P < 0.001; **P < 0.01; *P < 0.05

#### Statistically significant individual and institutional variables

In the analysis (results shown in Table 14), individual variables emerged as important and institutional variables emerged as unimportant (in terms of the occurrence and the size of the regression coefficients). What did not enter the equation? Age, being a female academic, holding parallel jobs, holding full professorship, and attaching importance to one’s academic discipline and academic satisfaction. Also in the block of “socialization to academia,” both variables related to doctoral studies are statistically insignificant. In the internationalization and collaboration in research block, two variables (international collaboration and publishing abroad) statistically significantly increase the odds of becoming a top performer. Domestic collaboration in research does not enter the equation and “research international in scope or orientation” actually decreases the odds of entering the class of highly productive academics.

In the academic behaviors and working time allocation, annualized mean weekly research hours emerged as powerful determinative predictors of high research productivity: a 1-h unit increase (in annualized research hours per week) increases the odds of being a top performer by 3.8%, on average (*ceteris paribus*). In the academic attitudes and role orientation block, research orientation emerges as a powerful predictor, with Exp(B) = 2.333. In the inferential analyses and in the regression analyses, long research hours and high research orientation emerge as important characteristics of top performers. The variables related to the understanding of scholarship (scholarship is best defined as original research by the respondents) and to the characterization of one’s primary research as basic or theoretical did not enter the equation.

To strengthen the robustness of the logistic regression analysis, separate models for top performers from all academic fields (ALL), STEM academics, and SSH academics—as well as for top performers defined as the upper 5, 10, and 15% of academics in terms of their research productivity—were constructed (not shown here for space limitations). Overall, in these models in addition to the core STEM/10% model, new independent variables entered the equation only exceptionally. In the ALL/5% model, full professorship increases the odds twice (Exp(B) = 2.211), consistent with the accumulative advantage theory, and in the SSH/5% models, possessing only a PhD or lower degree decreases the odds three times (Exp(B) = 0.343). Both findings are consistent with the traditional seniority-based structure of the Polish academic profession in which research funding opportunities have been opened to younger academics only within the last few years, following the creation of the National Research Council (NCN) in 2010 (Kulczycki et al. 2017; Kwiek 2017b).

In the ALL/10% model, intensive faculty guidance increases the odds almost six times (Exp(B) = 5.837), and being a peer reviewer increases the odds four times (Exp(B) = 4.192). Two other independent variables emerge as more significant (research orientation and serving as a journal editor). Interestingly, international research orientation emerges as a powerful predictor of being a top performer (Exp(B) = 5.511). The only difference between the core STEM/10% model and the SSH/10% model is the emergence in the equation of only two variables, albeit with lower intensity. For SSH academics, the only two predictors are international collaboration in research (Exp(B) = 3.569) and publishing abroad (Exp(B) = 5.84). In statistical terms, nothing else matters—which is a good lesson for new entrants in the profession in this cluster on the one hand, and for national and institutional academic career policies. Finally, in the case of models for top performers defined more widely, in the ALL/15% model a new variable enters the equation: sitting in international committees and boards (Exp(B) = 4.759). For STEM and SSH academics, the predictors are the same, with slightly different intensities.

## Discussion and conclusions

The Polish academic profession, despite functioning in the last three decades in largely different conditions from the academics traditionally studied in research productivity literature (see Pinheiro and Antonowicz 2015; Siemieńska and Walczak 2012; Wolszczak-Derlcz and Parteka 2010; Kwiek 2015b), follows the same stratification pattern: the tiny minority of 10% (termed top performers) produces about half of all Polish academic knowledge production. Without top performers, the Polish academic knowledge production would be halved. Kyvik (1989: 209) came to similar conclusions about the skewness of Norwegian productivity (the most prolific 20% of the faculty produced 50% of the total research output) and Abramo et al. (2009: 143) presented similar findings about Italian productivity patterns (12% of authors accounted for 35% of the total research output, averaged among the disciplinary areas). However, what would happen to Polish science *without* the remaining 90% of academics is unknown: the old question (Gasset 1932) to what extent non-publishing authors and low publishing authors (as well as uncited publications) contribute to scientific progress was beyond the scope of this paper. Therefore the issues to study in the future are the dependence of eminent scientists in their work on mediocre scientists—and the reliance of top scientists on other top scientists only, as citation patterns may indicate; see the Ortega hypothesis analyzed in Seglen (1992) and Cole and Cole (1973: 216–234).

This research shows that consistently across major clusters of academic disciplines, top performers produce about half (44.7%) of all Polish publications (as well as 48.0% of publications in English and 57.2% of internationally co-authored publications). Their mean research productivity across major clusters is much higher (on average, 7.3 times) than that of the other academics, and in terms of internationally co-authored publications, it is, on average, 12.07 times higher. Strong cross-disciplinary differences are observed, however. For instance, top performers in humanities produce, on average, 60.5% of all internationally co-authored publications, and in medicine and health-related fields, as much as 70.5%.

Interestingly, the average research productivity distribution is highly skewed (with a long tail on the right) not only for all Polish academics in the sample, which could have been expected, but also for its segment of top performers. The upper 10% of academics is as internally stratified as the lower-performing 90%, with a very small number of very high publishers: the right tail of the productivity distribution tends to behave exactly as the entire productivity distribution. This result is consistent with recent findings by Yair et al. (2017: 5) who showed in a sample of Israel Prize laureates that the tail of excellence may behave as the entire productivity distribution. In a similar vein, Abramo et al. (2017a: 334) found the same pattern in the Italian national research system: “research productivity distribution for all fields is highly skewed to the right, both at overall level and within the upper tail”. This is also the case in Poland.

The bivariate analysis section of this paper showed that the stronger research role orientation of top performers is statistically significant, as is the stronger teaching role orientation among the rest of academics (following a long line of mostly survey-based research, Fox 1992; Ramsden 1994; Teodorescu 2000; Cummings and Finkelstein 2012; Jung 2014). Top performers value research: being interested primarily in teaching virtually excludes Polish academics from the class of top performers. International collaboration and publishing abroad significantly increase the odds of becoming a top performer (see Kwiek 2017c on ‘international research collaboration’ and ‘international research orientation’ across Europe). Annualized mean weekly research hours emerged as a powerful determinative predictor of high research productivity (in some clusters, for instance in physical sciences and mathematics, on average, top performers spend an additional 75 full working days per year, or 13 h per week, on research, which is the entry ticket to the highly productive class of academics if academic careers are considered). A standard pattern for Polish top performers is (many) more working hours, and especially (many) more research hours, than the discipline average. Both in the inferential analyses and the regression analyses, long research hours and high research orientation emerge as important characteristics of Polish top performers, consistent with research literature.

Longer working hours, and especially longer research hours, substantially contribute to high productivity (as shown before in Jung 2014; Shin and Cummings 2010; Teichler et al. 2013). In more competitive Polish disciplines in which competitive funding is more widely available (such as life sciences or physical sciences and mathematics), top performers work many more hours compared to the average academics in these disciplines. However, in less competitive disciplines (such as humanities and social sciences, with marginal access to competitive research funding), the differences between the two groups are not statistically significant. Also in the logistic regression analysis, annualized mean weekly research hours emerged as powerful determinative predictors of high research productivity (consistent with Cummings and Finkelstein 2012: 58; Drennan et al. 2013: 127; Shin and Cummings 2010: 590).

The most instructive example comes from life sciences (with 422 cases and the highest number of statistically significant differences between the two subpopulations among several academic activities studied). The top performers in life sciences, on average, seem to follow all traditional accounts of productive academics in the sociology of science. On average, they work almost 7 more hours per week, and specifically, they have the traditional working time distribution attributed to high publishers (Fox 1983; Hagstrom 1974) according to which research-time allocations compete directly with teaching-time allocations (Fox 1992; Kyvik 1990; Ramsden 1994), or the only relevant difference is in general between research time and non-research time (Stephan 2012). Their average weekly teaching time is 3.5 h shorter, and their research time 4 h longer; in addition, they spend 3.7 more hours on administration (presumably more research involves more research grants which require more administrative work; alternatively, these academics are more often heads of research groups or medium-level administrators, such as directors and deans).

However, this research has its limitations. Three streams of research studied in literature could not be followed. First, it was not possible to study differences between top performers from institutions of lower academic standing and those from the most prestigious institutions, knowing that minor and major universities (as in Agrawal et al. 2017; Crane 1965) may provide more and less favorable academic settings and attract more and less talented students and academics, respectively. Location and affiliation may matter not only for recognition but also for high research productivity, which could not be verified with the dataset used. It could not be studied whether top performers gravitate toward institutions and departments in which research is a priority (as White et al. (2012) explored in a sample of business faculty). Neither within-department (and institution) nor between-department (or institution) variability could be studied, as in Perianes-Rodrigues and Ruiz-Castillo (2015) and in Toutkoushian et al. (2003).

Second, Polish top performers could not be linked to the 963 basic academic units periodically assessed by KEJN (Committee for the Evaluation of Scientific Units that uses national marks to assess the relative research level of each unit, which determines the level of public research subsidies for a period of 4 years; see Kulczycki 2017). For this reason, a study of the impact of highly productive academics on the general productivity of their academic units—or of the asymmetry of knowledge production between the within-unit top performers and the within-unit other academics across different institutions—could not be performed (following Piro et al. 2016 who studied Norwegian universities, with the conclusion that their overall productivity impact on units is modest). Top performers may increase the productivity of those present in the organization, and they may also increase the productivity of newly hired members due to their reputation (Agrawal et al. 2017). However, with the instrument used, this could not be explored. And third, only a cross-sectional study could be performed; thus, no changes over time could be analyzed (for instance, the identification of the persistence of top performance over time as in Kelchtermans and Veugelers (2013), or the length of periods of the stardom of stars as in Abramo et al. (2017b) could not be explored).

In logistic regression analysis, surprisingly in the context of much research productivity literature, in the block of personal and demographic variables, being a female academic did not enter the equation (thereby not confirming the results found in Abramo et al. 2009 about Italian ‘star scientists’). Abramo and colleagues (2009: 143) in their study of Italian ‘star scientists’ conclude that the star scientist “is typically a male full professor” and that female star scientists are primarily concentrated in the lesser levels of productivity. Holding a parallel academic job—contrary to expectations in a country with a large, albeit decreasing, private higher education sector—did not emerge as a predictor of not becoming a top performer. Surprisingly in the context of previous research on Poland (Antonowicz 2016; Antonowicz et al. 2017; Białecki and Dąbrowa-Szefler 2009), in two complementary models constructed specifically for social scientists and humanists (the SSH/5% and SSH/15% models), holding a parallel job (in research institutes or higher education institutions) actually increases the odds of high research performance. However, these two models do not pertain to the research productivity of the Polish academic profession in general, only to very high research productivity of its social sciences and humanities segment. Overall, the combination of all models shows similar predictors of entering the class of Polish top performers. Also attaching importance to one’s academic discipline (as opposed to one’s academic institution), traditionally dividing more productive cosmopolitans from less productive locals (with fundamentally different frames of reference in conducting research and publishing research results, leading them to seeking different sources of recognition and to having different trajectories of academic careers; across Europe, see Kwiek 2017c; Wagner and Leydesdorff 2005) and satisfaction with one’s current job did not enter the equation (as in Teichler et al. 2013).

While, similarly to most studies (Crane 1965; Drennan et al. 2013; Postiglione and Jung 2013), age did not emerge as a statistically significant variable, also holding full professorship or having a Habilitation degree in the Polish case (both representing academic seniority) had no statistical significance. Being a senior-ranking faculty did not increase the odds of becoming a top performer. This finding does not confirm the conclusions from previous productivity studies and highlights the specificity of the Polish academic career. A good explanation can be that Polish academics are not more likely to be promoted to higher ranks if they are highly productive. High research productivity in Poland does not seem to affect promotion to full professorship. Also intensive faculty guidance and research projects conducted with faculty during doctoral studies are statistically insignificant (inconsistent with findings in Horta and Santos (2016) who focused on the impact of publishing during doctoral studies on future productivity). Unfortunately, the following could not be tested: a long line of research in which current affiliation matters (through contacts or halo effects), whether graduates of major universities are more likely to be highly productive than graduates of minor universities, and whether the next generation’s most productive scientists come from a highly selected group of previous top scientists (Crane 1965).

Consistent with previous research (Bentley 2015; Marquina and Ferreiro 2015; Shin and Cummings 2010; Kwiek 2016a), international collaboration and publishing abroad statistically significantly increase the odds of becoming a top performer. However, as Ramsden (1994: 223) argued, “identifying correlates of high productivity does not mean that we have identified causal relations”. Domestic collaboration in research does not enter the equation. “Research international in scope or orientation” (as an academic attitude) actually decreases the odds of entering the class of Polish highly productive academics, contrary to studies that tend to suggest a close correlation between internationalization understood as collaboration in research (as an academic behavior) and research productivity. There are at least two possible explanations for this finding. First, the international scope or orientation in research does not have to imply international research collaboration (and does not have to be linked to international publishing). Second, Polish academics may tend to view international research orientation through the lenses of European Union (EU) collaborative research projects and EU structural funds for research, which are often focused on international collaboration rather than on highly competitive research leading to top-tier publications.

The determinative power of institutional-level predictors emerged as marginal, consistent with previous research on productivity (Cummings and Finkelstein 2012: 59; Ramsden 1994: 220; Shin and Cummings 2010: 588; Teodorescu 2000: 212). While Drennan and colleagues (2013: 128) concluded in a cross-national study that “institutional factors were found to have very little impact on research productivity,” the present study results suggest these factors have zero impact. This finding is also consistent with the conclusion about the American professoriate that intrinsic motivations rather than institutional incentive structures (Finkelstein 2006: 97–98; Teodorescu 2000: 217) stimulate research productivity. This might mean that, generally, neither institutional policies nor institutional support matters substantially in becoming a top performer in Poland, possibly because top performers and low performers are scattered across the whole system.

Finally, the paper shows that global patterns of stratification in science—found in the classical sociology of science and in recent bibliometric studies—hold firmly in a heavily under-resourced and vertically undifferentiated Polish higher education system.^1^ Polish academic knowledge production is highly skewed and does not follow a normal distribution. In this sense, the production is undemocratic and follows a Paretian (power law) distribution. In a system currently rapidly changing into a much more competition-based one, inequalities in research productivity are only beginning to lead to inequalities in resources and rewards, potentially with new haves being recruited from top performers and new have-nots from low performers.

## Data Appendix

See Tables 15, 16, 17, 18, 19, 20, 21, 22, 23 and Figs. 8 and 9.

**Table 15:** Clusters of personal and institutional characteristics linked to individual research productivity, formulations of the relevant survey questions

*Personal/demographics*
Female (Question F1): “What is your gender?”
Mean age (Question F2): Calculated from “Year of birth”
Full-time (Question A7): “How is your employment situation in the current academic year at your higher education institution/research institute?”
PhD or lower degree (Question A1): “What is your academic rank?”
Habilitation degree (Question A1): “What is your academic rank?”
Full professorship (Question A1): “What is your academic rank?”
Work at another research institute or HEI (Question A8): “Do you work for an additional employer or do additional remunerated work in the current academic year?—In addition to your current employer, you also work at another research institute or higher education institution”
Self-employed (Question A8): “Do you work for an additional employer or do additional remunerated work in the current academic year?—In addition to your current employer, you are also self-employed”
My academic discipline/field is important (Question B4): “Please indicate the degree to which each of the following affiliations is important to you—My academic discipline/field” (answers 1 and 2 on five-point Likert scale from “very important” to “not important at all”)
My institution is important (Question B4): “Please indicate the degree to which each of the following affiliations is important to you—My institution” (answers 1 and 2 on five-point Likert scale from “very important” to “not important at all”)
Satisfaction with current job (Question B6): “How would you rate your overall satisfaction with the current job”? (answers 1 and 2 on five-point Likert scale from “very high” to “very low”)
*Socialization to academia*
Intensive faculty guidance (Question A3): “How would you characterize the training you received in your doctoral degree?—You received intensive faculty guidance for your research”
Research projects with faculty (Question A3): “How would you characterize the training you received in your doctoral degree?—You were involved in research projects with faculty or senior researchers”
*Internationalization and collaboration*
Collaborating internationally (Question D1): “How would you characterize your research efforts undertaken during this (or the previous) academic year?—Do you collaborate with international colleagues?”
Collaborating domestically (Question D1): “How would you characterize your research efforts undertaken during this (or the previous) academic year?—Do you collaborate with persons at other institutions in your country?”
Publishing in a foreign country (Question D5): “Which percentage of your publications in the last 3 years were published in a foreign country?”
Research international in scope or orientation (Question D2): “How would you characterize the emphasis of your primary research this (or the previous) academic year?”
*Academic behaviors*
Annualized mean research hours (60% in session and 40% not in session) (Calculated from Questions B1/1 and B1/2): “Mean weekly research hours (in session)” and “Mean weekly research hours (not in session): “Considering all your professional work, how many hours do you spend in a typical week on each of the following activities?”; “Research (reading literature, writing, conducting experiments, fieldwork”
*Academic attitudes and role orientation*
Research-oriented (Question B2): “Regarding your own preferences, do your interests lie *primarily* in teaching or in research?”; answer 4 only: “Primarily in research”
Basic/theoretical research (Question D2): “How would you characterize the emphasis of your primary research this (or the previous) academic year?—Basic/theoretical”
*Overall research engagement*
National/international committees/boards/bodies (Question A13): “During the current academic year, have you done any of the following?—Served as a member of national/international scientific committees/boards/bodies”
A peer reviewer (Question A13): “During the current academic year, have you done any of the following?—Served a peer reviewer (e.g. for journals, research sponsors, institutional evaluations)”
Editor of journals/book series (Question A13): “During the current academic year, have you done any of the following?—Served as an editor of journals/book series”
*Institutional policies*
Strong performance orientation (Question E4): “At my institution there is…—A strong performance orientation”
Research considered in personnel decisions (Question E6): “To what extent does your institution emphasize the following practices?—Considering the research quality when making personnel decisions”
*Institutional support*
Availability of research funds (Question B3): “At this institution, how would your evaluate each of the following facilities, resources, or personnel you need to support your work?—Research funding”
Supportive attitude of administration (Question E4): “At my institution there is…—A supportive attitude of administrative staff towards teaching/research activities”

**Table 16:** Research productivity: peer-reviewed articles (PRA) published in the 3-year reference period, research top performers (10%) versus the rest (90%)

	Rest (90%)								Top performers (upper 10%)					
	Mean PRA	95% confidence interval, lower band	95% confidence interval, upper band	Median	% non-publishers	Mean PRA for publishers only	Standard Deviation	*N*	Mean PRA	95% confidence interval, lower band	95% confidence interval, upper band	Median	Standard Deviation	*N*
HUM	2.42	2.11	2.73	0.5	42.7	6.19	3.67	551	13.37	11.47	15.27	13	7.62	62
SOC	2.95	2.41	3.49	0	47.2	7.61	4.47	262	13.67	10.67	16.67	14	8.24	29
PHYSMATH	3.24	2.64	3.84	2	39.7	7.66	4.02	174	16.64	14.23	19.05	16	5.49	20
LIFE	2.45	2.07	2.83	0	51.2	8.46	3.82	380	17.68	15.52	19.84	16	7.55	47
ENGITECH	2.03	1.75	2.31	0	52	7.5	3.28	511	16.83	14.79	18.87	15.3	8.08	60
AGRICULT	2.28	1.75	2.81	0	49.6	6.92	3.49	164	13.57	9.66	17.48	11.2	8.69	19
MEDHEALTH	1.95	1.55	2.35	0	55.5	7.74	3.39	282	16.77	13.56	19.98	17	9.11	31

**Table 17:** Research productivity: internationally co-authored peer-reviewed article equivalents (IC-PRA) published in the 3-year reference period, research top performers (10%) versus the rest (90%)

	Rest (90%)							Top performers (upper 10%)						
	Mean IC-PRA	95% confidence interval, lower band	95% confidence interval, upper band	Median	% *not* intern. co-authoring	Standard Deviation	*N*	Mean IC-PRA	95% confidence interval, lower band	95% confidence interval, upper band	Median	% *not* intern. co-authoring	Standard Deviation	*N*
HUM	0.06	0.02	0.1	0	92.2	0.44	551	0.74	0.03	1.45	0	77.1	2.81	62
SOC	0.07	0.03	0.11	0	92.8	0.34	262	0.52	− 0.08	1.12	0	87.0	1.58	29
PHYSMATH	1.18	0.81	1.55	0	63.2	2.48	174	8.23	5.07	11.4	9	10.7	6.76	20
LIFE	0.6	0.41	0.79	0	74.6	1.86	380	5.88	3.59	8.16	2.84	20.8	7.79	47
ENGITECH	0.24	0.16	0.32	0	82.3	0.92	511	2.61	1.45	3.78	0.75	44.7	4.51	60
AGRICULT	0.23	0.08	0.38	0	83.8	0.98	164	0.99	0.15	1.82	0	52.7	1.74	19
MEDHEALTH	0.21	0.08	0.34	0	86.4	1.11	282	2.54	0.83	4.25	0.15	49.4	4.67	31

**Table 18:** Research productivity: English language peer-reviewed article equivalents (ENG-PRA) published in the 3-year reference period, research top performers (10%) versus the rest (90%)

	Rest (90%)						Top performers (upper 10%)							
	Mean ENG-PRA	95% confidence interval, lower band	95% confidence interval, upper band	Median	% *not* publishing in English	Standard Deviation	*N*	Mean ENG-PRA	95% confidence interval, lower band	95% confidence interval, upper band	% *not* publish. in English	Median	Standard Deviation	*N*
HUM	0.57	0.44	0.7	0	63.2	1.54	551	3.48	2.22	4.74	23.8	1.55	4.96	62
SOC	0.55	0.34	0.75	0	62.9	1.64	262	2.55	1.02	4.08	25.6	1.05	4.03	29
PHYSMATH	3.09	2.5	3.69	1	40.3	3.99	174	16	13.32	18.68	0	16	5.72	20
LIFE	2	1.65	2.35	0	53.3	3.46	380	14.22	11.74	16.69	0	13.5	8.43	47
ENGITECH	1.29	1.07	1.52	0	56.7	2.58	511	10.58	8.59	12.57	4.3	9.9	7.71	60
AGRICULT	1.39	0.97	1.8	0	53.1	2.66	164	5.81	3.06	8.55	0	4.28	5.7	19
MEDHEALTH	1.02	0.75	1.28	0	60.6	2.24	282	9.14	6.2	12.08	6.8	8	8.02	31

**Table 19:** The total number of publications—as measured by ‘peer-reviewed articles (PRA), ‘peer-reviewed article equivalents’ (PRAE), ‘internationally co-authored article equivalents’ (IC-PRAE), and ‘English language article equivalents’ (ENG-PRAE)—published in the 3-year reference period, by top performers and the rest, by clusters of academic disciplines

	By top performers (upper 10%)	By the rest (90%)	Total published	By top performers (*n* %)
HUM				
PRAE	1969.9	3048.0	5017.9	39.3
IC-PRAE	95.6	62.4	158.0	60.5
EPRAE	483.2	631.1	1114.3	43.4
PRA	829.5	1319.6	2149.1	38.6
SOC				
PRAE	1052.3	1593.9	2646.1	39.8
IC-PRAE	41.4	43.8	85.2	48.6
ENG-PRAE	167.9	259.3	427.2	39.3
PRA	389.6	754.3	1143.9	34.1
PHYSMATH				
PRAE	414.3	652.3	1066.6	38.8
IC-PRAE	190.9	213.9	404.8	47.2
ENG-PRAE	392.4	593.2	985.6	39.8
PRA	331.0	564.4	895.4	37.0
LIFE				
PRAE	1286.6	1227.9	2514.5	51.2
IC-PRAE	404.8	267.1	671.9	60.3
ENG-PRAE	1003.9	930.2	1934.0	51.9
PRA	829.3	925.6	1754.9	47.3
ENGITECH				
PRAE	1610.5	1714.7	3325.2	48.4
IC-PRAE	262.5	178.1	440.7	59.6
ENG-PRAE	992.0	976.3	1968.3	50.4
PRA	1015.1	1029.9	2045.0	49.6
AGRICULT				
PRAE	505.9	525.0	1030.9	49.1
IC-PRAE	38.9	57.5	96.4	40.4
ENG-PRAE	214.0	302.6	516.6	41.4
PRA	264.0	371.7	635.7	41.5
MEDHEALTH				
PRAE	963.1	893.8	1856.8	51.9
IC-PRAE	151.9	63.6	215.5	70.5
ENG-PRAE	519.9	399.3	919.2	56.6
PRA	521.7	541.8	1063.4	49.1
Total				
PRAE	7802.5	9655.5	17,458.0	44.7
IC-PRAE	1186.0	886.4	2072.5	57.2
ENG-PRAE	3773.3	4091.9	7865.2	48.0
PRA	4180.2	5507.1	9687.4	43.2

**Table 20:** All Polish academics: the distribution of PRAE published in the 3-year reference period, by clusters of academic fields and publication number groups (in percent)

	(0,10)	<10,20)	<20,30)	<30,40)	<40, 50)	<50, inf)
HUM	52.8	28.9	12.2	3.8	1.5	0.8
SOC	38.5	38.8	13.7	5.6	1.7	1.7
PHYSMATH	69.3	23.2	7.0	0.5	0.0	0.0
LIFE	58.5	24.3	10.4	5.4	0.9	0.5
ENGITECH	60.8	25.4	9.3	3.4	0.6	0.5
AGRICULT	63.4	25.4	5.0	4.3	1.3	0.6
MEDHEALTH	61.6	20.3	10.3	4.6	2.1	1.2

**Table 21:** Top performers: the distribution of PRAE published in the 3-year reference period, by clusters of academic fields and publication number groups (in percent)

	0	(0,10)	<10,30)	<30,40)	<40, 50)	<50, inf)
HUM	0.0	0.0	60.8	24.4	9.7	5.1
SOC	0.0	0.0	46.1	33.3	10.3	10.4
PHYSMATH	0.0	0.0	97.1	2.9	0.0	0.0
LIFE	0.0	0.0	69.0	24.4	4.2	2.4
ENGITECH	0.0	0.0	78.0	16.7	2.9	2.5
AGRICULT	0.0	0.0	68.9	21.3	6.7	3.1
MEDHEALTH	0.0	0.0	62.0	22.1	10.0	6.0

**Table 22:** Various personal and institutional characteristics linked to high individual research productivity, research top performers versus the rest of academics (frequencies in percent or averages)

Items	Top performers (upper 10%)	Rest (90%)	|*Z*| or |*t*|* statistics	*P* value	Group with sig. higher proportion/mean
Female	35.98	45.51	2.90	0.004	Rest
Mean age	50.19	45.82	5.94*	< 0.001	Top
Full-time	99.44	98.55	1.46	0.144	
Professor	33.30	13.64	8.35	< 0.001	Top
Intensive faculty guidance	46.94	54.22	2.30	0.022	Rest
Research projects with faculty	45.40	45.08	0.07	0.947	
Collaborating internationally	81.13	63.73	5.60	< 0.001	Top
Collaborating domestically	70.51	48.67	6.73	< 0.001	Top
Publishing in a foreign country	49.14	14.35	14.08	< 0.001	Top
Research international in scope	52.31	40.74	3.42	0.001	Top
Mean research hrs (in session)	20.67	16.98	5.12*	< 0.001	Top
Mean res. hrs (not in session)	27.41	24.21	4.72*	0.004	Top
Research-oriented (answer 4)	17.92	12.50	2.53	0.011	Top
Research-oriented (answers 3 & 4)	84.29	67.43	5.61	0.000	Top
Research reinforces teaching	34.28	39.93	1.77	0.077	
Scholarship is original research	60.41	61.09	0.21	0.834	
Basic/theoretical research	45.80	29.64	4.95	< 0.001	Top
National/international committees	90.02	74.02	5.37	< 0.001	Top
A peer reviewer	16.45	10.92	2.37	0.018	Top
Editor of journals/book series	74.11	66.94	2.35	0.019	Top
Writing research grants	56.24	57.00	0.21	0.830	
Strong performance orientation	55.20	55.18	0.03	0.974	
Research in personnel decisions	13.14	9.27	2.03	0.042	Top
Availability of research funds	57.70	58.59	0.29	0.774	
Availability of research equipment	53.84	60.91	2.14	0.032	Rest
Availability of research laboratories	20.14	22.17	2.49	0.013	Rest
Supportive attitude of administration	35.98	45.51	2.53	0.011	Rest

**Table 23:** An inverse correlation matrix: diagonal values

Variable	Diagonal value
Female	1.097
Age	2.042
Full-time	1.022
PhD or lower	2.328
Full professorship	1.701
Intensive faculty guidance	1.064
Research projects with faculty	1.109
Collaborating internationally	1.187
Collaborating domestically	1.518
Publishing in a foreign country	1.553
Published abroad	1.358
Research international in scope or orientation	1.300
Mean research hours	1.206
Research-oriented	1.114
Scholarship is original research	1.056
Basic/theoretical research	1.079
National/international committees/boards/bodies	1.171
A peer reviewer	1.311
Editor of journals/book series	1.057
Strong performance orientation	1.054
Performance-based resource allocation	1.104
Availability of research funds	1.081
Supportive attitude of administration	1.068
Work at another research institute or higher education institution	1.041
Self-employed	1.042
My academic discipline/field is important	1.058
My institution is important	1.124
Satisfaction with current job	1.163

## Footnotes

1 *Main Science and Technology Indicators* (OECD 2017) show that Gross Domestic Expenditure on R&D (GERD) as a percentage of Gross Domestic Product (GDP) for Poland was the second-lowest in the European Union in 2015 (1.00, the lowest being for Greece with 0.97; 1.96 for EU-28 countries, 2.10 for EU-15 countries, and 2.38 for OECD countries). Also Higher Education Expenditure on R&D (HERD) as a percentage of Gross Domestic Product (GDP) for Poland was the third-lowest in the European Union in 2015 (0.29, higher only to Hungary, 0.17, and Slovenia, 0.23; 0.45 for EU-28 countries, 0.48 for EU-15 countries, and 0.42 for OECD countries).
